# Genetic Regulation of Chlorophyll Biosynthesis in Pepper Fruit: Roles of *CaAPRR2* and *CaGLK2*

**DOI:** 10.3390/genes16020219

**Published:** 2025-02-13

**Authors:** Huagang Sun, Yiyue Zhang, Lingkui Zhang, Xiang Wang, Kang Zhang, Feng Cheng, Shumin Chen

**Affiliations:** 1 Engineering Laboratory of Genetic Improvement of Horticultural Crops of Shandong Province, College of Horticulture, Qingdao Agricultural University, Qingdao 266109, China; sunhuagang1206@163.com; 2 State Key Laboratory of Vegetable Biobreeding, Key Laboratory of Biology and Genetic Improvement of Horticultural Crops of the Ministry of Agriculture and Rural Affairs, Institute of Vegetables and Flowers, Chinese Academy of Agricultural Sciences, Beijing 100081, China; zhangyiyue127@163.com (Y.Z.); zhanglk960127@163.com (L.Z.); wangxiang961001@163.com (X.W.); zhangkang01@caas.cn (K.Z.); chengfeng@caas.cn (F.C.)

**Keywords:** pepper fruit color, chlorophyll, genetic mapping, bulked segregant analysis (BSA)

## Abstract

**Background:** Pepper (*Capsicum annuum* L.) is a widely cultivated vegetable crop worldwide, with its rich fruit colors providing unique visual traits and economic value. This study investigated the genetic basis of the immature green fruit color by constructing a F_2_ segregating population derived from a cross between yellow fruit C20 and green fruit C62 parent lines. **Methods:** Bulked segregant analysis sequencing (BSA-seq) was performed to identify genomic regions associated with fruit color. Candidate genes were pinpointed through functional annotation and genetic variation analysis, supported by SNP markers, genotype analysis, and transcriptome profiling. **Results:** Two genomic regions associated with fruit color were identified on chromosomes 1 (14.55–20.85 Mb) and 10 (10.15–22.85 Mb), corresponding to previously reported loci *pc1* and *pc10.1*. Two chlorophyll synthesis-related genes, *CaAPRR2* and *CaGLK2*, were identified as candidate regulators of fruit color. Mutations in these genes include a premature stop codon in both *CaGLK2* and *CaAPRR2*. The mutation of *CaAPRR2* and *CaGLK2* jointly regulate the yellow fruit trait in pepper, with *CaGLK2* being the major gene and *CaAPRR2* being the minor gene. Transcriptome analysis showed that the expression levels of the two genes increased during the green ripening stage of the parent fruits, with higher expression levels of *CaGLK2*. **Conclusions**: This study identifies *CaGLK2* and *CaAPRR2* as key regulators of immature green fruit color in pepper, with *CaGLK2* playing a predominant role. These findings provide a theoretical foundation and data support for elucidating the molecular regulatory mechanisms of fruit color and advancing marker-assisted breeding in pepper.

## 1. Introduction

Pepper (*Capsicum annuum* L.) is an important vegetable crop widely used for food and seasoning purposes [1]. Beyond its use as a fresh vegetable and condiment, pepper is also a significant industrial raw material with applications in the food, chemical, and agricultural industries [2]. Originating from Central and South America, it belongs to the Solanaceae family [3]. One of the most crucial commodity qualities of pepper fruit is its color, which reflects its maturity and nutritional diversity. Pepper fruit color varies widely, including red, yellow, orange, green, purple, and more. The study of color variation in peppers holds significant importance due to its implications for market value, consumer preferences, and nutritional content. As color can act as a visual marker of fruit quality, understanding the underlying genetic mechanisms is essential for enhancing fruit quality through breeding programs.

The color of pepper fruit depends on the relative content of chlorophyll, carotenoid, anthocyanin, and other pigments [4]. Chlorophyll, anthocyanin, and carotenoid in pepper fruit have specific antioxidant effects, reducing the risk of inflammation and cardiovascular diseases [5]. The diverse colors of pepper fruit have made it a model plant for studying fruit color inheritance [6].

Chlorophyll, essential for photosynthesis in autotrophic plants, includes chlorophyll a and b, which bind to proteins on thylakoid membranes in chloroplasts to capture light, transfer energy, and initiate charge separation [7]. Chlorophyll content in fruits influences nutrition and flavor, while promoting chloroplast development enhances photosynthetic efficiency and sugar accumulation [8]. Chlorophyll biogenesis and development require coordinated activity between chloroplast and nuclear genes [9].

Mutant analyses in plants like tomatoes have identified several plastid and nuclear-encoded genes involved in chloroplast biogenesis and chlorophyll metabolism [10], including *SIMYB72* [11], *SGR1* [12], *APRR2-Like* [13], *GLK2* [14], and *LOL1* [15]. The downregulation of *SIMYB72* in tomatoes leads to uneven fruit coloration and increased chlorophyll accumulation, along with enhanced photosynthetic rate. *SGR1* is a key gene regulating chlorophyll degradation; its knockout in tomatoes through gene editing results in homozygous mutants where chlorophyll degradation is inhibited, leading to a green fruit phenotype upon ripening. In peppers, the stay-green phenotype is primarily attributed to a single nucleotide mutation in the *CaSGR1* gene, which significantly impacts fruit pigment content and the expression of related genes. This finding underscores the importance of *CaSGR1* in regulating chlorophyll retention and pigment dynamics during fruit maturation [16].

In cucumbers, a premature stop codon in the *APRR2-like* gene leads to the white coloration of immature fruits, highlighting its critical role in regulating chlorophyll accumulation during fruit development [17]. In bitter melons, a premature stop codon in the *APRR2-like* gene was identified through BSA-seq analysis, resulting in the development of white fruit peels. Overexpression of the gene in tomatoes has been shown to result in the green pigmentation in the peels of immature fruits, further supporting its role in regulating chlorophyll accumulation [18]. The *APRR2-Like* gene, a positive regulator of chlorophyll synthesis in tomatoes, was also cloned in peppers, where a single base substitution in the white-fruited pepper introduced a premature stop codon, limiting chlorophyll accumulation [19].

The *GLK2* gene, a nuclear-encoded transcription factor from the *GARP* family, plays a crucial role in regulating chloroplast development and chlorophyll biosynthesis. In *Camellia sinensis* (tea plant), *CsGLK* transcription factors have also garnered significant attention for their regulatory role under UV-B radiation, enhancing chlorophyll biosynthesis and promoting flavonoid accumulation [20] promotes chloroplast development by activating genes encoding photosynthetic proteins, resulting in deeper green fruit coloration [21]. The pepper ortholog *CaGLK2* has been identified as a candidate gene for the *pc10.1* locus. Previous studies have shown that a single nucleotide mutation in this gene introduces a premature stop codon, truncating the protein translation to only 163 amino acids, which is associated with reduced chlorophyll levels in pepper fruits [22,23]. Variations in *CaGLK2* alleles contribute to a gradient of green shades in fruit, with certain alleles resulting in lighter green phenotypes [24].

The *LOL1* gene in pepper encodes a zinc finger transcription factor that plays a critical role in fruit development within the Solanaceae family. *LOL1* impacts chlorophyll content by regulating genes involved in photosynthesis and redox processes. In tomato, knockout studies of *LOL1* homologs revealed a light green fruit phenotype, confirming its role in chlorophyll regulation and suggesting that *LOL1* affects the stability of chlorophyll and the photosynthetic apparatus [25].

The *CaPP2C35* gene was identified as a regulator of light-green immature fruit color through GWAS and BSA-seq analysis. The gene was shown to influence chlorophyll biosynthesis and plastid development, with its mutation leading to decreased chlorophyll accumulation [26].

Allelic variations in APRR2-*Like*, *GLK2*, and *LOL1* in immature peppers result in changes in fruit chlorophyll content, resulting in diverse color phenotypes ranging from light green to dark green and even yellow [27]. This variation provides insights into the genetic mechanisms of fruit coloration in peppers, offering potential targets for future breeding efforts to develop pepper varieties with enhanced coloration traits.

In this study, we constructed a F_2_ segregating population by crossing green fruit pepper C62 and yellow fruit pepper C20, and used bulked segregant analysis sequencing (BSA-seq) [28] to identify candidate genes. We analyzed the genotypes of *CaLOL1*, *CaAPRR2*, and *CaGLK2* in an additional set of 258 pepper cultivars. The results indicate that mutations in *CaAPRR2* and *CaGLK2* are significantly associated with the yellow fruit color phenotype. Subsequent phenotypic and genotypic analyses, together with transcriptomic analysis, supported the regulatory roles of these candidate genes in determining fruit color. The results of this study can be applied in molecular marker-assisted breeding to develop pepper varieties with desirable color characteristics, enhancing their appearance, nutritional content, and commercial value. This research provides a foundational reference for further studies on fruit quality and edible value, promoting sustainable and efficient production in the pepper industry.

## 2. Materials and Methods

### 2.1. Experimental Materials

In this study, we used two pepper varieties as parental materials: ‘Meiren Xiaocaijiao’ pepper C20 (with yellow fruit during the mature green stage and red mature fruit, female parent) and ‘Zunla-1’ pepper C62 (with dark green fruit during the mature green stage and red mature fruit, male parent). The experimental materials were provided by the Bioinformatics Research Group of the Institute of Vegetables and Flowers, Chinese Academy of Agricultural Sciences.

### 2.2. Population Construction

The parental materials were hybridized in the spring of 2021 at the Institute of Vegetables and Flowers, Chinese Academy of Agricultural Sciences, resulting in 8 F_1_ plants. In the autumn of 2021, the F_1_ plants were self-pollinated to obtain F_2_ seeds. In the spring of 2022, a F_2_ segregating population was constructed, with a total of 423 F_2_ plants. In July 2022, all F_2_ pepper fruits were collected during the mature green stage and classified according to the color grades. The fruits from both the parental lines and the F_2_ population (three fruits per individual plant) were evaluated using PhenoAI [29], and the color of the F_2_ fruits was classified by combining this with visual classification. The RGB values of each fruit were extracted using the software, and the green intensity was quantified using the formula 2G−R−B, where G, R, and B represent the green, red, and blue color components, respectively. Higher values of this index indicate a greater degree of greenness. This approach provided an objective and quantitative measurement to complement the visual classification.

### 2.3. Chlorophyll Content Determination

To assess chlorophyll content in the fruit pericarps of C20 and C62 at three different developmental stages: 1, 7, and 21 days after flowering (designated as 1 DAF, 7 DAF, and 21 DAF, respectively). We collected three biological replicates for each stage. Approximately 0.1 g of finely chopped fresh fruit pericarp samples were placed in 50 mL centrifuge tubes, and 95% ethanol was added to a final volume of 50 mL. The tubes were tightly wrapped with aluminum foil and placed on a shaker at 50 rpm for approximately 48 h, or until the pericarps became white and devoid of any green color. The supernatant extract was then poured into a cuvette, with 95% ethanol used as the blank control. Absorbance was measured at wavelengths of 649 nm and 665 nm and recorded [30].

The chlorophyll content was calculated using the following equations [31]:

Chlorophyll a (Ca) = 13.95D665 − 6.8D649

Chlorophyll b (Cb) = 24.96D649 − 7.32D665

Total chlorophyll (Ct) = Ca + Cb = 18.16D649 + 6.63D665

The pigment content was determined using the following formula:

Chlorophyll pigment content = C × V × D/W

C = Pigment concentration (mg/L)

V = Volume of extract (L)

D = Dilution factor

W = Sample fresh weight (g)

### 2.4. DNA Pool Construction and Library Preparation and Sequencing

Bulked segregant analysis (BSA-Seq) was performed using four DNA samples: two parental samples and two F_2_ segregation bulks. The parental DNA samples, designated P1 and P2, were derived from the male parent C62 and the female parent C20, respectively. For the segregation bulks, the D1 bulk was constructed by pooling genomic DNA from 13 F_2_ individuals exhibiting the extreme yellow fruit phenotype, while the D2 bulk comprised DNA from 20 F_2_ individuals with the extreme green fruit phenotype. Sequencing libraries were prepared according to the standard Illumina protocol and sequenced on the Illumina HiSeq™ 2500 platform (lumina, San Diego, CA, USA) [32] at BGI in Shenzhen, China. Raw sequencing data were converted into raw reads using Illumina Casava 1.8 software [33], followed by quality filtering to generate high-quality clean reads.

### 2.5. Read Alignment and Variant Calling

The filtered reads were aligned to the Zunla-1_v3.0 reference genome [34] using the Burrows–Wheeler Aligner (BWA v0.7.17) [35]. To minimize biases, SAMtools v1.17 [36] was used to remove duplicate reads and mask PCR duplicates. Subsequent local realignment and base quality recalibration were conducted using the Genome Analysis Toolkit (GATK) [37]. Single nucleotide polymorphisms (SNPs) and small insertions/deletions (indels) were identified and annotated with SnpEff 3.0 software [38].

### 2.6. Association Mapping Analysis and Candidate Region Identification

Two computational approaches were applied to identify genomic regions associated with fruit color: the Euclidean distance (ED) algorithm and the SNP/InDel index method. (1) The ED algorithm quantified allele frequency differences between the parental samples (P1 and P2) and the segregation bulks (D1 and D2) for each SNP and InDel [39]. DNA nucleotide frequencies (A, C, G, T) were used as indicators. ED values were squared to amplify significant signals and smoothed using LOESS regression for visualization and candidate region identification. (2) The ΔSNP/InDel index was calculated through the following steps: Retain only loci that were homozygous but differed between the two parents. Calculate the SNP/InDel index for each locus by determining the proportion of C62 genotypes within the segregation bulks. Derive the ΔSNP/InDel index by subtracting the SNP/InDel index of D2 from D1 at each locus, taking the absolute value [40]. Compute the average ΔSNP/InDel index for sliding windows of 3 Mb with a 300 kb step [41]. Smoothed ΔSNP/InDel index values were fitted using sliding window, and candidate regions were identified where values exceeded a threshold set at the top 1% of sliding window’s values. This threshold was adjusted as needed to capture biologically relevant regions [42,43,44].

### 2.7. Transcriptome Sequencing and Analysis

To investigate transcriptomic changes, fruit pericarps samples were collected from the yellow parent C20 and green parent C62 on 1, 4, 7, and 21 DAF. Each time point included three biological replicates, resulting in 24 transcriptome samples. Raw transcriptome sequencing data underwent quality control using fastp [44] to ensure high data reliability. Clean reads were aligned to the Zunla-1_v3.0 reference genome using HISAT2 [45], achieving alignment rates between 97.68% and 98.77% (Table S1). These high alignment rates confirm the suitability of using Zunla-1_v3.0 as a reference genome and indicate high-quality sequencing data. Gene expression levels were quantified using FeatureCounts [46], which generated a comprehensive gene expression matrix. Differentially expressed genes (DEGs) between the two parental lines at each time point were identified using DESeq2 (R4.1.2) [47]. This analysis provided insights into the transcriptional differences associated with fruit color development at different stages.

### 2.8. SNP Markers Development

To develop SNP markers for genotyping, candidate genes were analyzed for base mutations present in the parental lines. Primers were designed to amplify fragments containing the mutation sites, ensuring that the target mutation was located at least 100 bp from the left primer, with an amplicon size ranging from 300 to 600 bp. These SNP markers were validated through Sanger sequencing to confirm genotypes [48]. The resulting sequence data were analyzed using Geneious Prime version 2023.2.1 to identify and quantify SNP variations [49]. Marker M1-9380, M1-9790 and M10-3560 were used to genotype *CaAPRR2*, *CaLOL1*, and *CaGLK2,* respectively. Detailed primers information is provided in Table S2.

## 3. Results

### 3.1. Inheritance of the Pepper Immature Fruit Color

**Figure 1:**
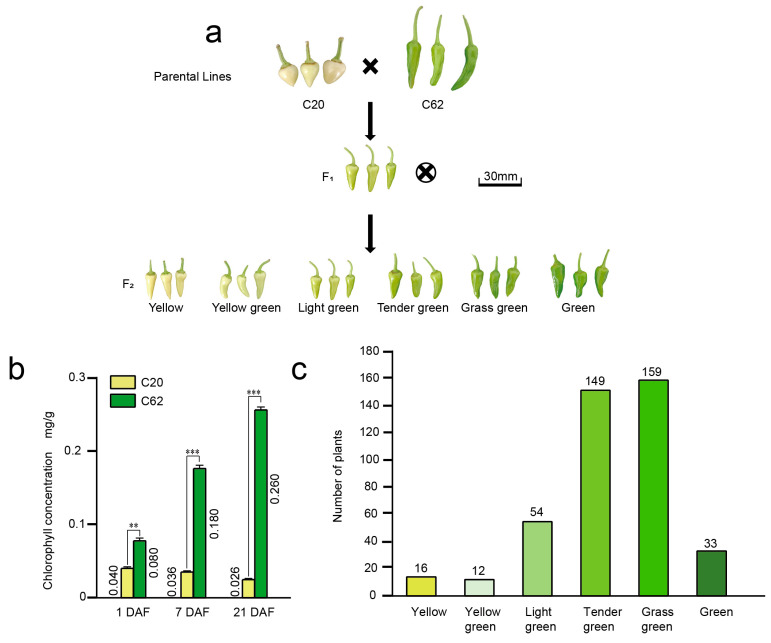
Segregation and chlorophyll content analysis in pepper fruits derived from a cross between parental lines C20 and C62. (**a**) Schematic representation of the cross between the yellow-fruited C20 and the green-fruited C62 parental lines, showing the F₁ generation and the color variation in the F₂ generation fruits (from yellow to green). Scale bar = 30 mm. (**b**) Chlorophyll content measured in fruits of C20 and C62 at three developmental stages (1, 7, and 21 DAF), showing a significant increase in chlorophyll levels in C62 compared to C20. Statistical significance is indicated (** *p* < 0.01; *** *p* < 0.001). (**c**) Distribution of fruit color phenotypes in the F₂ population, with a range of chlorophyll-related color classes from yellow to green.

The parental lines exhibited distinct fruit color during the mature green stage: C20 produced yellow, heart-shaped fruit, while C62 developed green, finger-shaped fruit. To understand the physiological basis of the observed fruit color differences, chlorophyll content in the pericarp of C20 and C62 was measured at three developmental stages: 1,4, 7 DAF, revealing significant differences across all stages. At 1 DAF, C62 exhibited a chlorophyll content of approximately 0.080 mg/g, compared to 0.040 mg/g in C20 (*p* =0.0060). By 7 DAF, the chlorophyll content in C62 increased to 0.180 mg/g, while C20 remained at 0.036 mg/g (*p* =0.0005). At 21 DAF, C62 reached 0.260 mg/g, nearly tenfold higher than C20 (0.026 mg/g) (*p* =8.7173 × 10^−5^) (Figure 1b). These results suggest that C62 has a superior capacity for chlorophyll accumulation, particularly during later stages of fruit development. To understand the genetic of chlorophyll content, we construct a F_2_ segregating population by crossing yellow pepper C20 and green pepper C62. Statistical analysis of fruit color in the F_2_ population during the mature green stage showed a normal distribution (Shapiro–Wilk (S–W) test, W = 0.810, *p* = 0.072) [50] (Figure 1a and Table 1), indicating that the null hypothesis was not rejected, and the results followed a normal distribution. Phenotype survey of the F_2_ population revealed six distinct fruit color categories: yellow, yellow-green, light green, tender green, grass green, and green, with counts of 16, 12, 54, 149, 159, and 33, respectively (Figure 1c). These findings demonstrate that green fruit color in peppers is a quantitative trait controlled by multiple genes.

### 3.2. Identifying Candidate Intervals for pc1 and pc10.1 Using BSA

We used BSA-Seq to identify the genetic loci of fruit color. To ensure the accuracy of the BSA, the variant dataset was filtered to include only sites that were homozygous in both parents but differed between them. After filtering, 8,956,124 SNPs/InDel variant sites remained for BSA. Using the genotype of the green parent as a reference, the proportion of this genotype in both pools was calculated as the SNP-index for each pool, and a sliding window analysis (window size: 3 Mb, step size: 300 kb) was performed across the genome. This revealed two significant association peaks located on chromosomes 1 and 10, with sufficient marker density (more than 10,000 markers per window) to minimize the likelihood of false positives.

**Figure 2:**
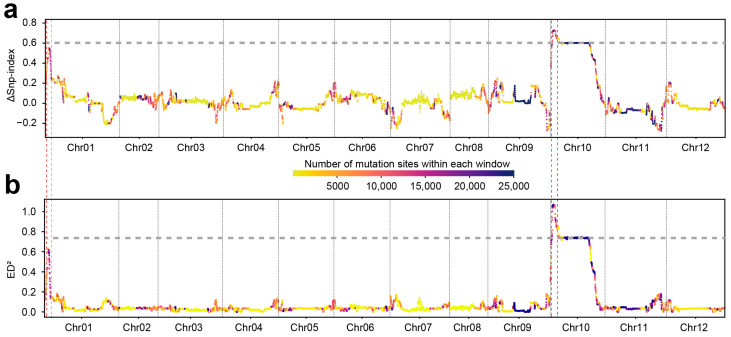
Genomic analysis of mutation sites across pepper chromosomes. (**a**) ΔSNP-index distribution across all 12 chromosomes, showing the relative differences in single nucleotide polymorphisms (SNPs) between two contrasting pepper lines. The x-axis represents chromosome position, while the y-axis shows ΔSNP-index values. The color gradient represents the number of mutation sites within each window, from yellow (lower mutation count) to dark blue (higher mutation count). Horizontal dashed lines indicate the significance threshold, and vertical dashed red lines highlight candidate regions with notable ΔSNP-index peaks. (**b**) ED^2^ distribution across all 12 chromosomes, indicating genetic differentiation between the two pepper lines. The x-axis represents chromosome position, while the y-axis represents ED^2^ values. The color gradient follows the same scale as in (**a**). Horizontal dashed lines represent the significance threshold, while vertical dashed red lines mark candidate regions with significant ED^2^ peaks.

At the association peaks on chromosomes 1 and 10, the genotypes in the yellow pool were derived from the yellow parent (SNP-index value >98%). In the green pool, some genotypes inherited from the yellow parent, with approximately 33% on chromosome 1 and approximately 26% on chromosome 10 (Figure S1). These findings suggest that green fruit color may be a dominant trait, with some dominant heterozygous loci present in the green pool. By calculating the ΔSNP-index between the pools, two candidate intervals were identified: 14.55–20.85 Mb on chromosome 1 and 10.15–22.85 Mb on chromosome 10 (Figure 2a).

Using the Euclidean Distance (ED) algorithm, the squared Euclidean Distance (ED^2^) values were calculated for the two pools, which corroborating the ΔSNP-index results. Two association peaks were confirmed on chromosomes 1 and 10, aligning with the intervals 14.55–20.85 Mb on chromosome 1 and 10.15–22.85 Mb on chromosome 10 (Figure 2b). These intervals correspond to previously reported loci: *pc1* on chromosome 1 and *pc10.1* on chromosome 10, which are associated with genetic control of pepper fruit color [27].

### 3.3. Identification of Key Chlorophyll Synthesis Genes CaAPPR2 and CaGLK2 by Functional Annotation and Parental Genetic Variation Analysis

This study aims to investigate the genes closely associated with the color differences between C20 and C62 peppers, with a particular focus on key loci located on chromosomes 1 and 10. The candidate interval on chromosome 1 (14.55–20.85 Mb) spans 6.3 Mb and includes 91 genes, 73 of which have functional annotations (Table S3). Among these, 34 genes exhibited coding region variations, including non-synonymous mutations, premature stop codons, and frameshift mutations (Table S4). On chromosome 10, the candidate interval (10.15–22.85 Mb) spans 12.7 Mb and encompasses 175 genes, with functional annotations for 157 of them (Table S5). Mutation and functional analyses identified 71 genes with significant variations (Table S6).

**Figure 3:**
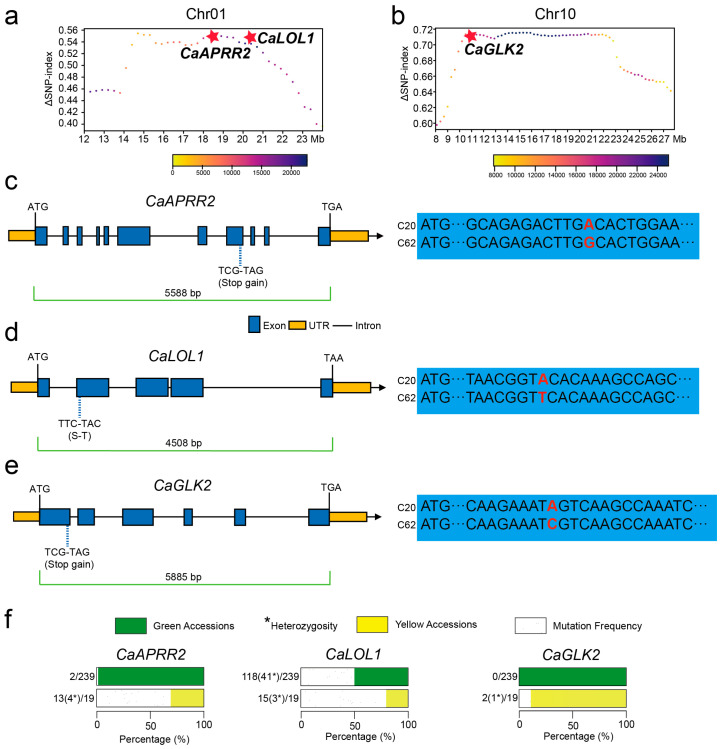
BSA mapping and structural analysis of candidate genes *CaAPRR2, CaLOL1*, and *CaGLK2* associated with chlorophyll synthesis in pepper fruit. (**a**) ΔSNP-index plot of chromosome 1 showing the position of the chlorophyll-related gene *CaAPRR2* and *CaLOL1*, marked by a red star. (**b**) ΔSNP-index plot of chromosome 10 indicating the location of the chlorophyll-related gene *CaGLK2*, also highlighted with a red star. (**c**) Structural diagram of *CaAPRR2* gene, showing a premature stop codon mutation (TGG to TGA) causing truncated protein synthesis. The right panel shows the sequence alignment of *CaAPRR2* in the C62 (green parent) and C20 (yellow parent) lines, with the mutation site highlighted. Blue boxes represent exons, yellow regions represent untranslated regions (UTRs), and black lines represent introns. The full length of the gene, including both introns and exons, is highlighted by a green line, spanning from the start codon to the stop codon. (**d**) Structural diagram of *CaLOL1* gene, highlighting a non-synonymous mutation (TTC to TAC) resulting in an amino acid change from serine (S) to tyrosine (T). (**e**) Structural diagram of *CaGLK2* gene, showing a premature stop codon mutation (TCG to TAG) causing truncated protein synthesis. The right panel presents the sequence alignment for *CaGLK2* in the two parental lines, highlighting the stop-gain mutation. (**f**) Genetic variation in CaAPRR2, *CaLOL1*, and *CaGLK2* in green and yellow accessions. The bar chart displays the genetic variation frequencies for three genes (*CaAPRR2*, *CaLOL1,* and *CaGLK2*) across green and yellow accessions, and heterozygosity is denoted by an asterisk (*). For *CaAPRR2*, yellow accessions exhibit a frequency of 13(4*)/19 (yellow bar), while green accessions show 2/239 (green bar). The mutation frequency is indicated as 2/239 (white bar for *CaLOL1*, green accessions have a frequency of 118(41*)/239, and yellow accessions exhibit a frequency of 15(3*)/19). *CaGLK2* shows no variation in green accessions (0/239), whereas yellow accessions display a frequency of 2(1*)/19. Data are derived from a total of 258 materials.

The color difference between the two parental lines is attributed to variations in chlorophyll content, prompting this study to focus on genes involved in chlorophyll biosynthesis. Additionally, certain genes on chromosomes 1 and 10 have been preliminarily implicated in chlorophyll biosynthesis, and thus, this study focuses on these candidate genes. Functional annotation of genes and genetic variations in the two genomic regions revealed three candidate genes associated with chlorophyll synthesis: *ZLC01G0009380* (*CaAPRR2*), *ZLC01G0009790* (*CaLOL1*), and *ZLC10G0003560* (*CaGLK2*). *CaAPRR2* is a transcription factor that regulates chlorophyll synthesis, and *CaLOL1*, a zinc finger transcription factor, plays a role in chlorophyll synthesis (Figure 3a), while *CaGLK2* is involved in regulating plastid spatial size in chloroplasts, and *CaCHLI1* is a magnesium chelatase subunit involved in chlorophyll synthesis, functioning as a key enzyme in the magnesium insertion step of the chlorophyll biosynthetic pathway (Figure 3b), in our population, *CaCHLI1* did not undergo any mutations. Notable genetic variations were observed between the green and yellow parent. In the green parent C62, all three genes exhibited normal structures. Conversely, in the yellow parent C20, mutations were identified in all three genes. For *CaAPRR2*, a single base mutation (guanine to adenine) at 1428 bp from the start codon resulted in a premature stop codon. This mutation is truncating the protein to 475 amino acids compared to the full-length 586 amino acids (Figure 3c). Notably, this mutation is consistent with the mutation reported in the *APRR2-like* gene on chromosome 8 in previously study, which indicates that it leads to the appearance of white fruit pericarp in peppers [19]. Interestingly, the same mutation is observed in different alleles, suggesting that these mutations may have a common origin. This highlights the functional significance of this site in determining fruit pericarp pigmentation and supports its role as a critical genetic determinant of the white fruit phenotype. For *CaLOL1*, a single base mutation (thymine to adenine) at 479 bp from the start codon resulted in a non-synonymous substitution (serine to tyrosine). For *CaGLK2*, a novel cytosine-to-adenine mutation at 247 bp from the start codon introduced a premature stop codon, truncating the protein to 81 amino acids compared to the full-length 313 amino acids (Figure 3d,e). The truncated *CaGLK2* protein in C20 was shorter than previously reported mutation, underscoring its pivotal role in regulating pepper fruit color. For *CaLOL1,* the non-synonymous mutation occurred outside the functional domain, so it does not affect the gene’s function. To determine whether the mutations in these three candidate genes are associated with green fruit color, we analyzed the genotype of three genes in 258 pepper materials, of which 19 exhibited yellow fruit. Genetic variation within this population revealed that the mutation in the *CaLOL1* gene was present in 133 materials, including 15 yellow fruit materials and 118 green fruit materials. This suggests that the mutation in *CaLOL1* is not associated with fruit color. For the *CaAPRR2* gene, 13 of the 19 yellow fruit materials carried the mutation, while 2 materials with green fruit carried the mutation. For the *CaGLK2* gene, two yellow fruit materials exhibited the same mutation site as C20 (Figure 3f). Therefore, we conclude that *CaAPRR2* and *CaGLK2*, but not *CaLOL1*, regulate green fruit color in pepper.

Additionally, several genes related to chlorophyll biosynthesis and pigment metabolism were identified on chromosomes 1 and 10 (Table S9). Among them, *CaCHLI1* is a magnesium chelatase subunit involved in chlorophyll synthesis, serving as a key enzyme in the magnesium insertion step of the chlorophyll biosynthetic pathway in our population [51]. Notably, *CaCHLI1* did not undergo any mutations and will not be included in further analysis.

### 3.4. Differential Expression of Photosynthesis-Related Genes During Fruit Development in Yellow and Green Pepper Lines

**Figure 4:**
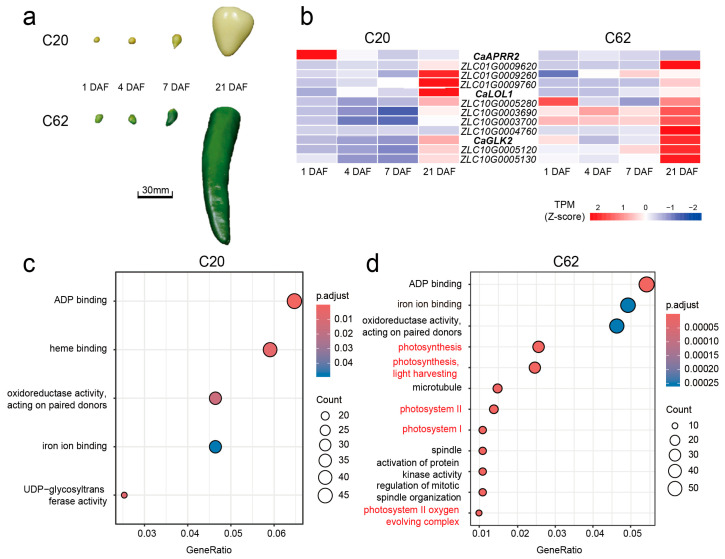
Comparative transcriptome and gene ontology enrichment analysis of parental lines C20 (Yellow parent) and C62 (Green parent) across four developmental stages. (**a**) Developmental stages of pepper fruit from parental lines C20 and C62, including 1, 4, 7, and 21 DAF. Scale bar = 30 mm. (**b**) Heatmap of transcript per million (TPM) expression values (Z-score) for candidate genes involved in chlorophyll biosynthesis and photosynthesis in C20 and C62 at four developmental stages. Higher expression levels are indicated in red, and lower levels in blue. (**c**) Gene ontology (GO) enrichment analysis of upregulated genes in C20, highlighting terms related to ADP binding, heme binding, oxidoreductase activity, iron ion binding, and UDP-glycosyltransferase activity. (**d**) GO enrichment analysis of upregulated genes in C62, with significant enrichment in terms related to photosynthesis, including photosystem I, photosystem II, and the photosystem II oxygen-evolving complex (in red), as well as additional molecular functions and cellular components associated with photosynthesis and cell organization. The size of each circle represents the number of genes in each category, and the color gradient indicates the adjusted p-value of enrichment significance.

To validate the impact of these genetic differences on fruit color, transcriptome sequencing was conducted on fruit pericarp from both parental lines at four developmental stages: 1, 4, 7, and 21 DAF (Figure 4a). Differential gene expression was assessed using HISAT2 for alignment and FeatureCounts for quantification, with TPM values calculated for normalization. Across all stages, *CaGLK2* exhibited higher expression levels in the green parent, while *CaAPRR2* showed relatively higher expression in the yellow parent (Figure 4b).

The heatmap visualization (Figure 4b) shows distinct expression dynamics for *CaAPRR2*, *CaLOL1*, and *CaGLK2* during fruit development in the two parental lines (C20 and C62). Specifically, *CaGLK2* and *CaLOL1* exhibit a gradual increase in expression from 1 DAF to 21 DAF, while *CaAPRR2* demonstrates a progressive decline over the same developmental stages, highlighting potential differences in their functional roles during fruit maturation. Transcriptomic GO enrichment analysis revealed distinct upregulated biological processes in the two lines. In C20, there were 24 upregulated DEGs (Table S7) were enriched in oxidoreductase activity, iron ion binding, UDP-glycosyltransferase activity, heme binding, and ADP binding (Figure 4c). In C62, there were 51 upregulated DEGs (Table S8) associated with photosystem I and II, the photosystem II oxygen-evolving complex, regulation of mitotic spindle organization, protein kinase activation, and photosynthesis pathways, including light harvesting (Figure 4d). Shared molecular functions included oxidoreductase activity and ADP binding, highlighting overlap alongside lineage-specific traits.

### 3.5. Joint Regulation of Fruit Color by CaAPRR2 and CaGLK2 Through Dominant and Additive Effects

Based on the variations in the candidate genes *CaAPRR2* and *CaGLK2* between C20 and C62, SNP markers were developed for genotyping. The genotypes of the *CaAPRR2* and *CaGLK2* mutant sites in randomly selected individual plants from the F_2_ generation were analyzed, allowing the determination of the genotype distribution across different color grades. For ease of statistical analysis, we designated the genotypes of the two genes *CaAPRR2* and *CaGLK2* in green pepper parent as *CaAPRR2^GG^* and *CaGLK2^GG^*, respectively, and the genotypes of the two genes *CaAPRR2* and *CaGLK2* in yellow pepper parent as *CaAPRR2^YY^* and *CaGLK2^YY^*, respectively, with the heterozygous genotypes designated as *CaAPRR2^GY^* and *CaGLK2^GY^*. The genotype frequencies of individuals in the F_2_ population were associated with the six color grades.

Analysis of genotype frequencies and phenotypes (Table 2 and Table 3) revealed clear patterns for *CaAPRR2* and *CaGLK2* in regulating fruit color. In the F_2_ population, extreme yellow phenotypes lacked the *CaAPRR2^GG^* genotype, while *CaAPRR2^GY^* predominated among intermediate types, and *CaAPRR2^YY^* was least frequent in extreme green phenotypes. For *CaGLK2*, *CaGLK2^GG^* genotype was absent in yellow and yellow-green fruits, but its frequency increased with greener fruit, while *CaGLK2^YY^* was absent in grass green and green grades.

These results highlight dominant effects: when *CaGLK2* is homozygous recessive (YY), fruit becomes progressively greener as the *CaAPRR2* genotype shifts from YY to GY to GG. Similarly, with *CaAPRR2^YY^*, a greener phenotype emerges as *CaGLK2* transitions from YY to GY to GG.

Moreover, the combined additive effect of these genes was evident. The combination of *CaAPRR2^GG^* and *CaGLK2^GG^* produced the extreme green fruit phenotype, indicating a synergistic interaction between these genotypes. Similarly, *CaAPRR2^GY^* combined with *CaGLK2^GY^* resulted in a greener phenotype compared to either gene in the GY state alone. These findings suggest that *CaGLK2* primarily regulates fruit color, while *CaAPRR2* acts as a minor gene, with their additive and dominant interactions collectively influencing the green fruit color trait in peppers.

**Figure 5:**
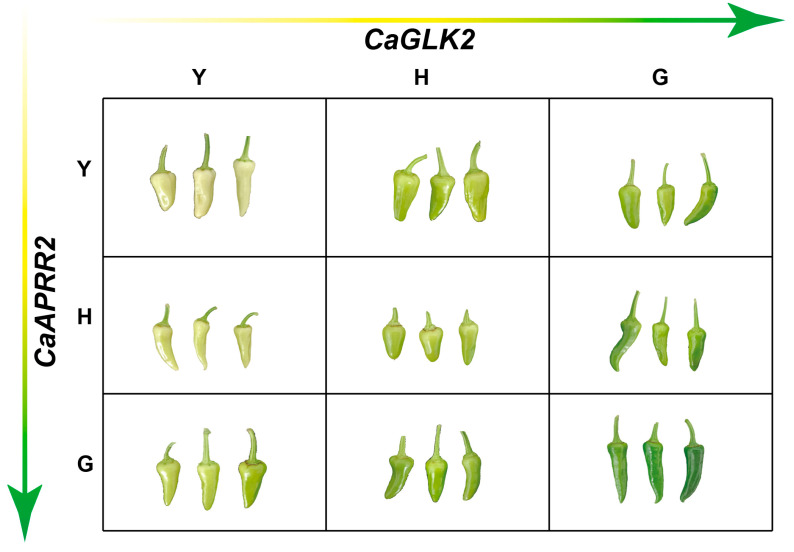
Effect of different genotypic combinations of candidate genes *CaAPRR2* and *CaGLK2* on pepper fruit color. Fruit color phenotypes for each genotype combination of *CaAPRR2* (Y = yellow homozygous, H = heterozygous, G = green homozygous) and *CaGLK2* (Y = yellow homozygous, H = heterozygous, G = green homozygous) are shown. The horizontal axis represents *CaGLK2* genotypes, and the vertical axis represents *CaAPRR2* genotypes. The gradient arrow illustrates the transition in fruit color from yellow to green as the genotypic combination shifts from YY to GG.

For genes *CaAPRR2* and *CaGLK2*, the genotypes of the green parent, heterozygous, and yellow parent are designated as G, H, and Y, respectively. The F_2_ population fruits are classified into nine categories based on genotypes (Figure 5), resulting in nine different colors of pepper fruits. When both genes are of the C20 yellow parent genotype, the color is yellow, consistent with the phenotype of the C20 yellow parent. As the genotype of the C62 green parent gradually increases, the color of pepper fruits deepens, and when both genes are of the C62 green parent genotype, the color is green, consistent with the phenotype of the C62 green parent.

On the other hand, different gene combinations have different effects on the biosynthesis of specific chlorophyll in pepper fruits. When the genotype of gene *CaGLK2* is Y and the genotype of gene *CaAPRR2* is G, H, or Y, the fruit color undergoes a slight change. However, when the genotype of gene *CaAPRR2* is Y and the genotype of gene *CaGLK2* is G, H, or Y, the change in fruit color is more pronounced, further confirming that gene *CaGLK2* is the major effective gene, while gene *CaAPRR2* is a minor gene.

## 4. Discussion

Fruit color is a critical trait of peppers, influencing consumer preferences, nutritional value, and commercial viability. To explore the genes regulating green fruit color traits in pepper, we constructed a F_2_ population using yellow pepper C20 and green pepper C62. The phenotypic and statistical analysis of the F_2_ population revealed a continuous distribution of fruit color, ranging from yellow to green. This suggests that green fruit color is influenced by multiple loci. BSA identified two candidate intervals on chromosome 1 (14.55 Mb–20.85 Mb) and chromosome 10 (10.15 Mb–22.85 Mb), corresponding to the *pc1* and *pc10.1* loci previously reported to regulate fruit color in peppers [27].

Additionally, we measured the chlorophyll content in the fruit pericarps of the parental lines, C20 and C62, at three developmental stages (1, 7, and 21 DAF). Significant differences in chlorophyll content between the parental lines, with C62 exhibiting higher chlorophyll levels at all stages compared to C20. This disparity in chlorophyll content correlates with the identified candidate intervals, suggesting that chlorophyll biosynthesis genes within these regions may influence the observed fruit color variations. These findings provide a biochemical basis for the observed phenotypic differences and support the genetic analysis of the candidate intervals.

Chlorophyll is the primary pigment for the green coloration in plant fruits that appear green. The *GLK2* gene encodes a pair of partially redundant nuclear transcription factors that influence chloroplast development [52]. The *APRR2* gene has been shown to be associated with the accumulation of chlorophyll in the fruit pericarp in both Cucurbitaceae and Solanaceae families. In this study, we identified three genes related to photosynthesis within the candidate intervals through functional annotation: *CaAPPR2* (*ZLC01G0009380*), *CaLOL1* (*ZLC01G0009790*), and *CaGLK2* (*ZLC10G0003560*). Variation analysis revealed that *CaAPPR2, CaLOL1*, and *CaGLK2* in the green parent exhibited normal structure and function. In contrast, in the yellow parent, *CaAPRR2* terminated prematurely, encoding only 475 amino acids, and *CaGLK2* terminated prematurely, encoding only 81 amino acids, suggesting that mutations in these genes significantly impact chlorophyll biosynthesis and fruit pigmentation. However, the *CaLOL1* gene exhibits a single nucleotide nonsynonymous mutation in a non-domain region, which does not appear to significantly impact the gene’s function. Through genotype analysis of 258 collected pepper accessions, we identified a widespread mutation at a specific site in the *CaAPRR2* gene, highlighting its critical role in chlorophyll accumulation in the fruit pericarp. In contrast, the mutation in the *CaGLK2* gene was rare and appeared to be restricted to two varieties, suggesting its unique genetic specificity. Meanwhile, the mutation in the *CaLOL1* gene, though present in some accessions, was not associated with chlorophyll accumulation in the fruit pericarp, indicating its negligible functional significance in this context.

Transcriptome analysis provided further insights into the functional roles of *CaGLK2* and *CaAPRR2* during fruit development. *CaGLK2* exhibited significantly higher expression in the green parent C62, particularly at later developmental stages, consistent with its role in promoting chloroplast development and chlorophyll accumulation. In contrast, *CaAPRR2* showed relatively higher expression in the yellow parent C20, though its mutation likely impairs its effectiveness in regulating chlorophyll biosynthesis. Gene Ontology (GO) enrichment analysis identified distinct biological processes associated with the two parental lines, with the green parent showing upregulation of photosynthesis-related pathways, including photosystem I and II, and light-harvesting processes. These results confirm the dominant role of *CaGLK*2 in regulating chlorophyll biosynthesis and highlight *CaAPRR2* as a secondary factor influencing chlorophyll content. Genotypic analysis of the F_2_ population revealed clear patterns of additive and dominant interactions between *CaGLK2* and *CaAPRR2*, which collectively regulate the gradient of fruit color from yellow to green. *CaGLK2* was identified as the primary regulator of chlorophyll accumulation, with its homozygous green genotype *CaGLK2^GG^* resulted in the deepest green fruit color. In contrast, *CaAPRR2* functioned as a minor gene, with its heterozygous and homozygous green genotypes *CaAPRR2^GY^* and *CaAPRR2^G^^G^* enhancing fruit color in combination with *CaGLK2*. Notably, the combination of *CaGLK2^GG^* and *CaAPRR2^GG^* resulted in the extreme green fruit phenotype, while the absence of functional *CaGLK2* (homozygous yellow genotype, *CaGLK2^YY^*) produced yellow fruit regardless of *CaAPRR2* genotype. These findings highlight the hierarchical relationship between these genes, with *CaGLK2* exerting a dominant effect on fruit color and *CaAPRR2* providing additional modulation through additive interactions.

## 5. Conclusions

In conclusion, our study identified two genes, *CaGLK2* and *CaAPRR2*, related to chlorophyll biosynthesis through BSA-seq analysis, revealing significant differences in chlorophyll content between the parental lines. We measured chlorophyll content in the fruit pericarps of the yellow pepper C20 and green pepper C62 at three developmental stages: 1, 7, and 21 DAF. The results indicated consistently higher chlorophyll levels in C62 compared to C20, particularly at the later stages of fruit development. Comprehensive analysis of phenotype and genotype determined *CaGLK2* as the major-effect gene and *CaAPRR2* as the minor gene, findings that were validated by temporal transcriptome analysis. These findings not only expand our understanding of the regulatory mechanisms underlying specific chlorophyll content in pepper fruits but also provide a solid foundation for further research in related fields.

## Figures and Tables

**Table 1:** Normality test analysis results.

Sample	Mean	SD	Skewness	Kurtosis	Shapiro–Wilk Test (W)	*p*-Value
Plants	70.500	66.431	0.775	−1.868	0.810	0.72 (ns)

1. Values are represented as mean ± standard deviation (n = 6). 2. *p*-value indicates the result of the Shapiro–Wilk test for normality. ns represents no significant difference (*p* > 0.05). The data follow a normal distribution based on this result.

**Table 2:** Individual plant genotype statistics of candidate gene *CaAPRR2*.

F_2_ generation Color Grading	*CaAPRR2^GG^*	*CaAPRR2^GY^*	*CaAPRR2^YY^*	Total
Yellow			9	9
Yellow green	2	2	5	9
Light green	5	19	6	30
Tender green	10	14	5	29
Grass green	6	16	6	28
Green	3	6	1	10

**Table 3:** Individual plant genotype statistics of candidate gene *CaGLK2*.

F_2_ generation Color Grading	*CaGLK2^GG^*	*CaGLK2^GY^*	*CaGLK2^YY^*	Total
Yellow		1	9	10
Yellow green		2	8	10
Light green	7	12	11	30
Tender green	6	20	3	29
Grass green	13	15		28
Green	7	3		10

## Data Availability

The original contributions presented in this study are included in the article/Supplementary Materials. Further inquiries can be directed to the corresponding author.

## Supplementary Materials

The following supporting information can be downloaded at: https://www.mdpi.com/article/10.3390/genes16020219/s1, Figure S1. SNP-index Distribution of Yellow and Green Mixing Pools Across Chromosomes; Table S1. Comparative filtering statistics analysis of transcriptome sequencing data; Table S2. Primer Information; Table S3. Annotated genes on Chromosome 1; Table S4. Mutation of Genes on Chromosome 1; Table S5. Annotated genes on Chromosome 10; Table S6. Mutation of Genes on Chromosome 10; Table S7. DEGs on Chromosome 1; Table S8. DEGs on Chromosome 10. Table S9. Genes related to the color of pepper fruits [53,54,55,56,57,58,59].
