# Supplementary material for: Genetic Regulation of Chlorophyll Biosynthesis in Pepper Fruit: Roles of CaAPRR2 and CaGLK2
Source: Genes (Basel). 2025 Feb 13;16(2):219. doi: 10.3390/genes16020219 (PMC11855580; doi:10.3390/genes16020219)
Supplement: Supplementary file 1 [file genes-16-00219-s001.zip › genes-3383842-supplementary/supplemental fugures.pdf]

Figure S1. SNP-index Distribution of Yellow and Green Mixing Pools Across Chromosomes

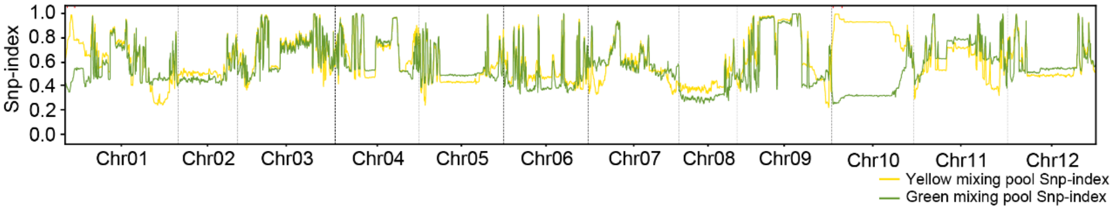

The figure illustrates the SNP-index distribution for the yellow mixing pool and green mixing pool across chromosomes (Chr01 to Chr12). The x-axis represents the chromosome numbers, and the y-axis indicates the SNP-index values ranging from 0 to 1. The variation in SNP-index along different chromosomes reflects the genomic diversity between the two pools.
