# Supplementary material for: Genetic Regulation of Chlorophyll Biosynthesis in Pepper Fruit: Roles of CaAPRR2 and CaGLK2
Source: Genes (Basel). 2025 Feb 13;16(2):219. doi: 10.3390/genes16020219 (PMC11855580; doi:10.3390/genes16020219)
Supplement: Supplementary file 1 [file genes-16-00219-s001.zip › genes-3383842-supplementary/supplemental tables.pdf]

Table S1. Comparative filtering statistics analysis of transcriptome sequencing data

	1 DAF		4 DAF		7 DAF		21 DAF	
	C20	C62	C20	C62	C20	C62	C20	C62
S1 reads	37,59<br>5,943	53,429<br>,418	39,581<br>,204	43,820<br>,439	40,15<br>7,798	50,07<br>3,943	40,40<br>2,728	40,24<br>2,340
Number of reads matched to the genome	38,17<br>8,<br>634	54,093<br>,<br>360	40,514<br>,<br>664	44,357<br>,<br>322	40,93<br>2,<br>250	50,62<br>9,<br>978	41,14<br>5,<br>150	40,79<br>5,<br>416
Ratios (%)	98.47	98.77	97.70	98.79	9801	98.90	98.20	98.64
S2 reads	40,77<br>6,<br>221	40,949<br>,<br>154	41,398<br>,<br>91	40,344<br>,<br>672	40,66<br>2,<br>317	43,27<br>1,<br>544	42,28<br>4,<br>865	39,02<br>9,<br>447
Number of reads matched to the genome	41,48<br>9,<br>920	41,492<br>,<br>708	42,318<br>,<br>790	40,867<br>,<br>310	41,38<br>9,<br>610	43,80<br>9,<br>618	43,05<br>2,<br>512	39,54<br>8,<br>278
Ratios (%)	98.28	98.69	97.83	98.72	98.24	98.77	98.22	98.69
S3 reads	41,47<br>5,<br>167	40,427<br>,<br>783	51,579<br>,<br>699	44,239<br>,<br>308	40,66<br>1,<br>838	40,68<br>9,<br>134	42,48<br>4,<br>050	42,79<br>6,<br>816
Number of reads matched to the genome	42,18<br>9<br>864	40,972<br>516	52,886<br>,<br>090	44,907<br>,<br>124	41,41<br>1,<br>648	41,24<br>8,<br>164	43,22<br>2,<br>768	43,31<br>2,<br>520
Ratios (%)	98.31	98.67	97.53	98.51	9809	98.64	98.29	98.81
average value (%)	98.35	98.71	97.68	98.67	9808	98.77	98.23	98.71

Table S2. Primer Information

Primer name	Forward primer	Reverse primer
M1-9380	CAAGGGAAGTGGAAAGGAGA	GAACTCAACCCAAGAGTGCAT
M1-9790	TAGTGGTACGGACTGTGTAC	TTGTGTTCATGCAGCTCTAC
M10-3560	GTTGTATCTACACCATTG	GATTACAAATGTCACGTG

Table S3. Annotated genes on Chromosome 1

Gene name	Annotation information
ZLC01G0008990	Intron maturase, type II family protein; (source: Araport11)
ZLC01G0009000	Encodes a member of the trxG protein family.
ZLC01G0009010	Plant invertase/pectin methylesterase inhibitor superfamily protein; (source: Araport11)
ZLC01G0009020	Pentatricopeptide Repeat Protein involved in splicing of nad 4, nad 5 and nad2 introns which affects biogenesis of the respiratory complex I.
ZLC01G0009030	Plant invertase/pectin methylesterase inhibitor superfamily protein; (source: Araport11)
ZLC01G0009040	pectin methylesterase inhibitor
ZLC01G0009050	Intron maturase, type II family protein; (source: Araport11)
ZLC01G0009060	Intron maturase, type II family protein; (source: Araport11)
ZLC01G0009080	Ubiquitin carboxyl-terminal hydrolase family protein; (source: Araport11)
ZLC01G0009100	Required for nonsense-mediated mRNA decay. Involved in RNA interference.
ZLC01G0009120	MraZ; (source: Araport11)
ZLC01G0009130	Encodes a member of the Lon protease-like proteins (Lon1/At5g26860, Lon2/At5g47040, Lon3/At3g05780, Lon4/At3g05790).
ZLC01G0009140	electron carrier/iron ion-binding protein; (source: Araport11)
ZLC01G0009150	Encodes a Protease inhibitor/seed storage/LTP family protein
ZLC01G0009160	encodes a gibberellin 20-oxidase. ARABIDOPSIS THALIANA GIBBERELLIN 20-OXIDASE 3; ATGA20OX3; GA20OX3; GIBBERELLIN 20-OXIDASE 3; YAP169
ZLC01G0009170	transducin family protein/WD-40 repeat family protein; (source: Araport11)
ZLC01G0009180	FAD-dependent oxidoreductase family protein; (source: Araport11)
ZLC01G0009200	SBP (S-ribonuclease binding protein) family protein; (source: Araport11)
ZLC01G0009210	Reticulon family protein; (source: Araport11)
ZLC01G0009220	hypothetical protein (DUF581); (source: Araport11)
ZLC01G0009230	pleckstrin homology (PH) domain-containing protein; (source: Araport11)
ZLC01G0009240	poly(U)-specific endoribonuclease-B protein; (source: Araport11)
ZLC01G0009250	centromere protein O; (source: Araport11)
ZLC01G0009260	Encodes a beta-amylase targeted to the chloroplast.
ZLC01G0009270	plant-specific SNARE located in cell plate of dividing cells.
ZLC01G0009280	Encodes a member of the RLCK VII-4 subfamily of receptor-like cytoplasmic kinases that has been shown to phosphorylate MAPKKK5

	Ser-599 and MEKK1 Ser-603, both players in PRR-mediated resistance to bacterial and fungal pathogens. PBL19; PBS1-LIKE 19
ZLC01G0009290	Involved in gene silencing. Locus-specific regulator of 24nt-siRNA expression, works together with CLSY1-4 as the master regulators of essentially all Pol-IV-dependent 24nt-siRNAs. ZLC31; ZLCOMATIN REMODELING 31; CLASSY3; CLSY3
ZLC01G0009300	Remorin family protein; (source: Araport11)
ZLC01G0009310	The gene encodes a MYB transcription factor belons to R2R3-MYB family of transcription factors. Knock-down mutant analysis indicates its role in root hair elongation. MAMYB; MEMBRANE ANCHORED MYB
ZLC01G0009320	allyl alcohol dehydrogenase-like protein; (source: Araport11)
ZLC01G0009330	encodes a novel protein involved in DNA repair from UV damage. Isolated by functional complementation of E. coli UV-sensitive mutants (UVR genes). DNA-DAMAGE-REPAIR/TOLERATION 101; DRT101
ZLC01G0009340	Vacuolar protein sorting 55 (VPS55) family protein; (source: Araport11)
ZLC01G0009350	SNARE-like superfamily protein; (source: Araport11)
ZLC01G0009360	Man1-Src1p-carboxy-terminal domain protein; (source: Araport11)
ZLC01G0009370	S-adenosyl-L-methionine-dependent methyltransferases superfamily protein; (source: Araport11)
ZLC01G0009380	Encodes pseudo-response regulator 2 (APRR2) that interacts with a calcium sensor (CML9). APRR2; PRR2; PSEUDO-RESPONSE REGULATOR 2
ZLC01G0009400	TOM2A encodes a 280 amino acid putative four-pass transmembrane protein with a C-terminal farnesylation signal, essential for efficient multiplication of tobacco mosaic viruses. TOBAMOVIRUS MULTIPLICATION 2A; TOM2A
ZLC01G0009420	Encodes an inositol polyphosphate 5-phosphatase that appears to have Type I activity. It can dephosphorylate IP3(inositol (1,4,5) P3) and IP4 (inositol (1,3,4,5) P4), but it does not appear to be active against phosphatidylinositol 4,5 bisphosphate. Overexpression of this gene renders plants insensitive to ABA in germination and growth assays. "5PTASE2; AT5PTASE2; INOSITOL (1,4,5) P3 5-PHOSPHATASE II; IP5PII; MYO-INOSITOL POLYPHOSPHATE 5-PHOSPHATASE 2"
ZLC01G0009430	Phosphoribosyltransferase family protein; (source: Araport11)
ZLC01G0009530	Transmembrane nitrate transporter. Involved in xylem transport of nitrate from root to shoot. Induced in response to high and low concentrations of nitrate. Not involved in nitrate uptake. Expressed in root pericycle cells under the control of MYB59. Also functions as a proton-coupled H+/K+ antiporter for K+ loading into the xylem.

	ATNPF7.3; NITRATE TRANSPORTER 1.5; NPF7.3; NRT1.5; NRT1/ PTR FAMILY 7.3
ZLC01G0009540	encodes a chloroplast pyruvate kinase beta subunit. The enzyme is less active than the other chloroplast pyruvate kinase beta subunit encoded by AT5G52920. Involved in seed oil biosynthesis. Can partially complement the AT5G52920 mutant. PKP3; PLASTIDIAL PYRUVATE KINASE 3
ZLC01G0009560	Transmembrane nitrate transporter. Involved in xylem transport of nitrate from root to shoot. Induced in response to high and low concentrations of nitrate. Not involved in nitrate uptake. Expressed in root pericycle cells under the control of MYB59. Also functions as a proton-coupled H+/K+ antiporter for K+ loading into the xylem. ATNPF7.3; NITRATE TRANSPORTER 1.5; NPF7.3; NRT1.5; NRT1/ PTR FAMILY 7.3
ZLC01G0009570	Member of the R2R3 factor gene family. ARABIDOPSIS MYB DOMAIN PROTEIN 111; ATMYB111; MYB DOMAIN PROTEIN 111; MYB111; PFG3; PRODUCTION OF FLAVONOL GLYCOSIDES 3
ZLC01G0009580	Encodes an ent-kaurenoic acid hydroxylase, a member of the CYP88A cytoZLCome p450 family. "CYTOZLCOME P450, FAMILY 88, SUBFAMILY A, POLYPEPTIDE 3; ATKAO1; CYP88A3; ENT-KAURENOIC ACID OXYDASE 1; KAO1"
ZLC01G0009590	encodes a protein whose sequence is similar to a 2-oxoglutarate-dependent dioxygenase The mRNA is cell-to-cell mobile.
ZLC01G0009600	2-oxoglutarate (2OG) and Fe (II)-dependent oxygenase superfamily protein; (source: Araport11)
ZLC01G0009610	LYR family of Fe/S cluster biogenesis protein; (source: Araport11) B14; NDUFA6
ZLC01G0009620	encodes a desulfoglucosinolate sulfotransferase, involved in the final step of glucosinolate core structure biosynthesis. Has a broad-substrate specificity with different desulfoglucosinolates, the best substrate is indole-3-methyl-dsGS, followed by benzyl-dsGS. Expression was induced by wounding, jasmonate and ethylene stimulates. ARABIDOPSIS SULFOTRANSFERASE 5A; ATSOT16; ATST5A; CORI-7; CORONATINE INDUCED-7; SOT16; SULFOTRANSFERASE 16
ZLC01G0009630	member of CYP709B "CYTOZLCOME P450, FAMILY 709, SUBFAMILY B, POLYPEPTIDE 1; CYP709B1"
ZLC01G0009640	2-oxoglutarate (2OG) and Fe (II)-dependent oxygenase superfamily protein; (source: Araport11)
ZLC01G0009650	2-oxoglutarate (2OG) and Fe (II)-dependent oxygenase superfamily protein; (source: Araport11)

ZLC01G0009660	ent-kaurenoic acid hydroxylase (KAO2) ARABIDOPSIS ENT-KAURENOIC ACID HYDROXYLASE 2; ATKAO2; CYP88A4; ENT-KAURENOIC ACID HYDROXYLASE 2; KAO2
ZLC01G0009670	NAD(P)-binding Rossmann-fold superfamily protein; (source: Araport11) ATSDR5; SDR5; SHORT-CHAIN DEHYDROGENASE REDUCTASE 5
ZLC01G0009680	MuDR family transposase; (source: Araport11)
ZLC01G0009690	Involved in the conversion of the early brassinosteroid precursor 24-methylenecholesterol to campesterol. Brassinosteroids affect cellular elongation. Mutants have dwarf phenotype. DWF1 is a Ca2+-dependent calmodulin-binding protein. CABBAGE 1; CBB1; DIM; DIM1; DIMINUTIA; DIMINUTO 1; DWARF 1; DWF1; ENHANCED VERY-LOW-FLUENCE RESPONSES 1; EVE1
ZLC01G0009710	encodes a protein with cytoZLCome P450 domain "CYTOZLCOME P450, FAMILY 87, SUBFAMILY A, POLYPEPTIDE 2; CYP87A2"
ZLC01G0009720	Encodes a protein most similar to the POLTERGEIST locus. Double mutant analysis of loss of function alleles indicates PLL1 functions redundantly with POL to regulate meristem size and pedicel length. Acts in a dose dependent manner with POL to suppress the clv1, clv2 and clv3 phenotypes. PLL1; POLTERGEIST LIKE 1
ZLC01G0009730	pentatricopeptide (PPR) repeat-containing protein; (source: Araport11) SOT1; SUPPRESSOR OF THYLAKOID FORMATION 1
ZLC01G0009740	Encodes ANAC071, a transcription factor involved in cell proliferation in incised inflorescence stems. ANAC071; NAC DOMAIN CONTAINING PROTEIN 71; NAC071
ZLC01G0009750	RING/U-box superfamily protein; (source: Araport11) PIR1; PP2CA INTERACTING RING FINGER PROTEIN 1
ZLC01G0009760	RING/U-box superfamily protein; (source: Araport11) PIR1; PP2CA INTERACTING RING FINGER PROTEIN 1
ZLC01G0009770	RING/U-box superfamily protein; (source: Araport11) PIR1; PP2CA INTERACTING RING FINGER PROTEIN 1
ZLC01G0009780	homologue of the animal Eyes Absent genes. encodes a tyrosine-specific phosphatase. the protein sequence lacks the cys-containing signature of the classical tyrosine phosphatases. belongs to the aspartate-based phosphatases. The enzyme activity is strictly metal-dependent. ATEYA; EYA; EYES ABSENT HOMOLOG
ZLC01G0009790	Encodes a protein with 3 plant-specific zinc finger domains that acts as a positive regulator of cell death. LOL1; LSD ONE LIKE 1
ZLC01G0009800	Encodes FdC2, a ferredoxin protein capable of alternative electron partitioning. FdC1 level increases in conditions of acceptor limitation at PSI. FDC2; FERREDOXIN C 2
ZLC01G0009810	Encodes LEA4-1, a member of the Late Embryogenesis Abundant (LEA) proteins which typically accumulate in response to low water availability conditions imposed during development or by the

	environment. ATLEA4-1; LATE EMBRYOGENESIS ABUNDANT 4-1; LEA4-1
ZLC01G0009820	phospholipid hydroperoxide glutathione peroxidase; (source: Araport11)
ZLC01G0009830	AT hook motif DNA-binding family protein; (source: Araport11) AHL13; AT-HOOK MOTIF NUCLEAR LOCALIZED PROTEIN 13
ZLC01G0009840	hypothetical protein; (source: Araport11) SAUR79; SMALL AUXIN UPREGULATED RNA 79
ZLC01G0009860	Direct target of AGAMOUS. Regulates patterning and differentiation of reproductive organs. AHL21; AT-HOOK MOTIF NUCLEAR LOCALIZED PROTEIN 21; GIANT KILLER; GIK
ZLC01G0009870	C2H2 domain regulatory protein. Functions downstream of GL2 during root hair development and regulates expression of targets RDH6, RSL2 and RSL4. EMB3022; EMBRYO DEFECTIVE 3022; ZINC FINGER PROTEIN 1; ZP1
ZLC01G0009880	NAOD encodes a functional acetylornithine deacetylase. Silenced lines plants flower early but have reduced fertility (siliques do not develop) as well as reduced ornithine levels.NAOD mediates a linear pathway for ornithine biosynthesis. ATNAOD; N2-ACETYLORNITHINE DEACETYLASE
ZLC01G0009890	2-oxoglutarate (2OG) and Fe (II)-dependent oxygenase superfamily protein; (source: Araport11)

Table S4. Mutation of Genes on Chromosome 1

Gene name	Mutation type
ZLC01G0008990	non synonymous mutation
ZLC01G0009000	non synonymous mutation
ZLC01G0009020	non synonymous mutation
ZLC01G0009030	frameshift mutation
ZLC01G0009040	non synonymous mutation
ZLC01G0009050	non synonymous mutation
ZLC01G0009080	non synonymous mutation
ZLC01G0009090	non synonymous mutation
ZLC01G0009100	non synonymous mutation
ZLC01G0009120	non synonymous mutation
ZLC01G0009130	non synonymous mutation
ZLC01G0009300	frameshift mutation
ZLC01G0009350	non synonymous mutation

ZLC01G0009370	non synonymous mutation
ZLC01G0009380	premature termination
ZLC01G0009390	structural variation
ZLC01G0009410	structural variation
ZLC01G0009440	non synonymous mutation
ZLC01G0009450	structural variation
ZLC01G0009480	non synonymous mutation
ZLC01G0009490	non synonymous mutation
ZLC01G0009500	structural variation
ZLC01G0009510	non synonymous mutation
ZLC01G0009520	structural variation
ZLC01G0009530	non synonymous mutation
ZLC01G0009550	structural variation
ZLC01G0009560	non synonymous mutation
ZLC01G0009610	non synonymous mutation
ZLC01G0009650	non synonymous mutation
ZLC01G0009660	non synonymous mutation
ZLC01G0009740	non synonymous mutation
ZLC01G0009790	non synonymous mutation
ZLC01G0009800	non synonymous mutation
ZLC01G0009810	non synonymous mutation

Table S5. Annotated genes on Chromosome 10

Gene name	Annotation information
ZLC10G0003470	kinase C substrate, heavy chain-like protein; (source: Araport11)
ZLC10G0003480	Encodes the OST3/6 subunit of the hetero-oligomeric plant oligosaccharyltransferase complex (OST). Also identified by GWAS as having a role in interspecific pollen tube recognition. ARTUMES; ATU; OLIGOSACCHARYLTRANSFERASE SUBUNIT 3/6; OST3/6
ZLC10G0003490	RING/U-box superfamily protein; (source: Araport11) ARABIDOPSIS TÃ³XICOS EN LEVADURA 41; ATL41
ZLC10G0003500	elongation factor P (EF-P) family protein; (source: Araport11)
ZLC10G0003510	elongation factor P (EF-P) family protein; (source: Araport11)
ZLC10G0003520	elongation factor P (EF-P) family protein; (source: Araport11)
ZLC10G0003530	elongation factor P (EF-P) family protein; (source: Araport11)

ZLC10G0003550	Member of a large family of putative ligands homologous to the Clavata3 gene. Consists of a single exon. ATCLE5; CLAVATA3/ESR-RELATED 5; CLE5
ZLC10G0003560	Encodes GLK1, Golden2-like 1, one of a pair of partially redundant nuclear transcription factors that regulate chloroplast development in a cell-autonomous manner. GLK2, Golden2-like 2, is encoded by At5g44190. GLK1 and GLK2 regulate the expression of the photosynthetic apparatus. ARABIDOPSIS GOLDEN2-LIKE 1; ATGLK1; GBF'S PRO-RICH REGION-INTERACTING FACTOR 1; GLK1; GOLDEN2-LIKE 1; GPRI1
ZLC10G0003570	encoding the RPN subunits of the 26S proteasome; The mRNA is cell-to-cell mobile. 26S PROTEASOME REGULATORY SUBUNIT S2 1A; ATRPN1A; RPN1A
ZLC10G0003590	Nucleic acid-binding, OB-fold-like protein; (source: Araport11)
ZLC10G0003600	Encodes a cytosolic L-tyrosine aminotransferase. AtTAT2 exhibits much broader amino donor specificity than AtTAT1 and can use not only Tyr but also Phe, Trp, His, Met, Leu, Ala, Ser, Cys, Asp, Asn, Gln, and Arg as amino donors. ATTAT2; TAT2; TYR AMINOTRANSFERASE 2
ZLC10G0003610	Confers auxin overproduction. Mutants have an over-proliferation of lateral roots. Encodes a C-S lyase involved in converting S-alkylthiohydroximate to thiohydroximate in glucosinolate biosynthesis. Induced in epidermal cells attacked by powdery mildew. The RTY enzyme is expected to function as a dimer (or a higher order multimeric complex), as all RTY-related enzymes with a defined crystal structure are known to form dimers or tetramers. ABERRANT LATERAL ROOT FORMATION 1; ALF1; HLS3; HOOKLESS 3; ROOTY; ROOTY 1; RTY; RTY1; SUPERROOT 1; SUR1
ZLC10G0003620	Homolog of vertebrate TPX2. Protein has three domains involved in nuclear targeting, one in nuclear export and two in microtubule binding. Involved in mitotic spindle assembly during late prophase and early prometaphase. ATTPX2; TARGETING PROTEIN FOR XKLP2; TPX2
ZLC10G0003630	Host immune receptor which recognizes the conserved effectors AvrE and HopAA1. CAR1; CEL-ACTIVATED RESISTANCE 1
ZLC10G0003650	Confers resistance to the biotrophic oomycete, Peronospora parasitica. Encodes an NBS-LRR type R protein with a putative amino-terminal leucine zipper. Fungal protein ATR13 induces RPP13 gene expression and disease resistance. The mRNA is cell-to-cell mobile. RECOGNITION OF PERONOSPORA PARASITICA 11; RECOGNITION OF PERONOSPORA PARASITICA 13; RPP11; RPP13
ZLC10G0003660	NB-ARC domain-containing disease resistance protein; (source: Araport11)

ZLC10G0003670	RNA-directed DNA polymerase (reverse transcriptase)-related family protein; (source: Araport11)
ZLC10G0003680	Encodes a canonical CC-type NLR protein that is required for the recognition of the T3SE HopZ1a as well as several other Hop effectors from the pathogenic bacteria P. syringae. HOPZ-ACTIVATED RESISTANCE 1; ZAR1
ZLC10G0003690	NB-ARC domain-containing disease resistance protein; (source: Araport11)
ZLC10G0003700	Disease resistance protein (CC-NBS-LRR class) family; (source: Araport11)
ZLC10G0003720	NB-ARC domain-containing disease resistance protein; (source: Araport11)
ZLC10G0003730	NB-ARC domain-containing disease resistance protein; (source: Araport11)
ZLC10G0003760	O-acyltransferase (WSD1-like) family protein; (source: Araport11)
ZLC10G0003780	basic helix-loop-helix (bHLH) DNA-binding superfamily protein; (source: Araport11)
ZLC10G0003790	basic helix-loop-helix (bHLH) DNA-binding superfamily protein; (source: Araport11)
ZLC10G0003800	Polynucleotidyl transferase, ribonuclease H-like superfamily protein; (source: Araport11)
ZLC10G0003820	Encodes alpha-2,4 tubulin. TUA2 and TUA4 encode identical proteins. The mRNA is cell-to-cell mobile. TUA2; TUBULIN ALPHA-2 CHAIN
ZLC10G0003860	HXXXD-type acyl-transferase family protein; (source: Araport11)
ZLC10G0003870	Encodes a malonyltransferase that may play a role in phenolic xenobiotic detoxification. The mRNA is cell-to-cell mobile. PHENOLIC GLUCOSIDE MALONYLTRANSFERASE 2; PMAT2
ZLC10G0003880	Encodes a malonyltransferase that may play a role in phenolic xenobiotic detoxification. The mRNA is cell-to-cell mobile. PHENOLIC GLUCOSIDE MALONYLTRANSFERASE 2; PMAT2
ZLC10G0003890	Alanyl-tRNA synthetase; (source: Araport11) ALANYL-TRNA SYNTHETASE; ALATS
ZLC10G0003900	HXXXD-type acyl-transferase family protein; (source: Araport11)
ZLC10G0003910	Encodes a flavin mononucleotide-binding flavodoxin-like quinone reductase that is a primary auxin-response gene. FLAVODOXIN-LIKE QUINONE REDUCTASE 1; FQR1
ZLC10G0003920	Encodes a cytosolic ascorbate peroxidase APX1. Ascorbate peroxidases are enzymes that scavenge hydrogen peroxide in plant cells. Eight types of APX have been described for Arabidopsis: three cytosolic (APX1, APX2, APX6), two chloroplastic types (stromal sAPX, thylakoid tAPX), and three microsomal (APX3, APX4, APX5) isoforms. At least part of the induction of heat shock proteins during light stress in Arabidopsis is mediated by H2O2 that is scavenged by

	APX1. Expression of the gene is downregulated in the presence of paraquat, an inducer of photoxidative stress. The mRNA is cell-to-cell mobile. APX1; ASCORBATE PEROXIDASE 1; ATAPX01; ATAPX1; CS1; MATERNAL EFFECT EMBRYO ARREST 6; MEE6
ZLC10G0003930	Encodes SPIKE1 (SPK1), the lone DOCK family guanine nucleotide exchange factor (GEF) in Arabidopsis. SPK1 is a peripheral membrane protein that accumulates at, and promotes the formation of, a specialized domain of the endoplasmic reticulum (ER) termed the ER exit site (ERES). SPK1 promotes polarized growth and cell-cell adhesion in the leaf epidermis. Mutant has seedling lethal; cotyledon, leaf-shape, trichome defects. SPIKE1; SPK1
ZLC10G0003940	DNAse I-like superfamily protein; (source: Araport11)
ZLC10G0003950	basic helix-loop-helix (bHLH) DNA-binding superfamily protein; (source: Araport11)
ZLC10G0003960	Protein kinase superfamily protein; (source: Araport11)
ZLC10G0003980	Pentatricopeptide repeat (PPR) superfamily protein; (source: Araport11)
ZLC10G0003990	Encodes a ubiquitin-specific protease. UBIQUITIN-SPECIFIC PROTEASE 20; UBP20
ZLC10G0004000	Encodes a mannanase belonging to clade 1 of the GH5 7 phylogenetic tree that exhibits high substrate affinity and catalytic efficiency on mannan substrates with main chains containing both glucose and mannose units such as konjac glucomannan and spruce galactoglucomannan. It is likely a glycoprotein. ATMAN2; ENDO-BETA-MANNASE 2; MAN2; MAN5-2
ZLC10G0004010	pyrroline-5-carboxylate reductase; (source: Araport11)
ZLC10G0004020	microtubule-associated protein; (source: Araport11)
ZLC10G0004030	MLP-like protein 31; (source: Araport11) MLP-LIKE PROTEIN 31; MLP31
ZLC10G0004040	Encodes a protein with 13.6% proline amino acids that is predicted to localize to the cell wall. The mRNA is cell-to-cell mobile. FLOE1
ZLC10G0004050	Member of the family of canonical mitochondrial DNA binding proteins. Single-stranded binding protein which does not interfere with MMEJ. MITOCHONDRIAL SINGLE-STRANDED BINDING PROTEIN 2; SSB2
ZLC10G0004060	hypothetical protein; (source: Araport11)
ZLC10G0004070	LLG2/3 are involved in regulation of pollen tube growth by trafficking of ANX/BUPS to the apical PM of pollen tubes. In the PM they function as co-receptors with ANX/BUPS to regulate ROS production. LLG2; LORELEI-LIKE-GPI ANCHORED PROTEIN 2
ZLC10G0004080	Encodes a malonyltransferase that may play a role in phenolic xenobiotic detoxification. The mRNA is cell-to-cell mobile. PHENOLIC GLUCOSIDE MALONYLTRANSFERASE 2; PMAT2
ZLC10G0004090	HXXXD-type acyl-transferase family protein; (source: Araport11)

ZLC10G0004100	Polynucleotidyl transferase, ribonuclease H-like superfamily protein; (source: Araport11) ORF120
ZLC10G0004110	HXXXD-type acyl-transferase family protein; (source: Araport11)
ZLC10G0004120	RNA polymerase II transcription mediator; (source: Araport11) MED17; MEDIATOR 17
ZLC10G0004130	nucleolar essential protein-like protein; (source: Araport11)
ZLC10G0004140	Encodes a redox activated co-chaperone, chloroplast localized thioredoxin, similar to prokaryotic types. ATHM2; THIOREDOXIN M2; TRXM2
ZLC10G0004160	RMA1 encodes a novel 28 kDa protein with a RING finger motif and a C-terminal membrane-anchoring domain that is involved in the secretory pathway. Has E3 ubiquitin ligase activity. ATRMA1; RING MEMBRANE-ANCHOR 1; RMA1
ZLC10G0004170	RMA1 encodes a novel 28 kDa protein with a RING finger motif and a C-terminal membrane-anchoring domain that is involved in the secretory pathway. Has E3 ubiquitin ligase activity. ATRMA1; RING MEMBRANE-ANCHOR 1; RMA1
ZLC10G0004180	RMA1 encodes a novel 28 kDa protein with a RING finger motif and a C-terminal membrane-anchoring domain that is involved in the secretory pathway. Has E3 ubiquitin ligase activity. ATRMA1; RING MEMBRANE-ANCHOR 1; RMA1
ZLC10G0004190	acyl-UDP-N-acetylglucosamine O-acyltransferase; (source: Araport11)
ZLC10G0004200	putative beta-expansin/allergen protein. Naming convention from the Expansin Working Group (Kende et al, 2004. Plant Mol Bio). Involved in the formation of nematode-induced syncytia in roots of Arabidopsis thaliana. ATEXPB3; ATHEXP BETA 1.6; EXPANSIN B3; EXPB3
ZLC10G0004210	F-box family protein; (source: Araport11)
ZLC10G0004220	F-box family protein; (source: Araport11)
ZLC10G0004230	Encodes a protein with reported similarity to GCR2 a putative G protein coupled receptor thought to be an ABA receptor. GCL2 also has similarity to LANCL1 and LANCL2, human homologs of bacterial lanthionine synthetase. GCL2; GCR2-LIKE 2
ZLC10G0004240	alpha/beta-Hydrolases superfamily protein; (source: Araport11)
ZLC10G0004250	Required for proper proliferation of basal cells. TWIN 2; TWN2; VALRS; VALYL TRNA SYNTHETASE
ZLC10G0004260	Required for proper proliferation of basal cells. TWIN 2; TWN2; VALRS; VALYL TRNA SYNTHETASE
ZLC10G0004270	alpha/beta-Hydrolases superfamily protein; (source: Araport11)
ZLC10G0004280	ELMO/CED-12 family protein; (source: Araport11)
ZLC10G0004290	hypothetical protein (DUF789); (source: Araport11)
ZLC10G0004300	early-responsive to dehydration stress protein (ERD4); (source: Araport11) GFS10; MTV14; OSCA40
ZLC10G0004310	Protein phosphatase 2C family protein; (source: Araport11)

ZLC10G0004320	Peroxisomal membrane 22 kDa (Mpv17/PMP22) family protein; (source: Araport11)
ZLC10G0004330	Pentatricopeptide repeat (PPR-like) superfamily protein; (source: Araport11)
ZLC10G0004340	Encodes a GH3-related gene involved in red light-specific hypocotyl elongation. Analysis of sense and antisense transgenic plants suggests that DFL2 is located downstream of redlight signal transduction and determines the degree of hypocotyl elongation. DFL2; DWARF IN LIGHT 2; GH3-10
ZLC10G0004350	Galactose oxidase/kelch repeat superfamily protein; (source: Araport11)
ZLC10G0004360	CTTNBP 2 amino-terminal-like protein; (source: Araport11) DEG23
ZLC10G0004370	Encodes a putative secretory carrier membrane protein (SC3). The mRNA is cell-to-cell mobile. ATSCAMP1; SC3; SCAMP1; SECRETORY CARRIER 3; SECRETORY CARRIER MEMBRANE PROTEIN 1
ZLC10G0004390	hAT dimerization domain-containing protein / transposase-like protein; (source: Araport11)
ZLC10G0004400	root UVB sensitive protein (Protein of unknown function, DUF647); (source: Araport11) ROOT UV-B SENSITIVE 6; RUS6
ZLC10G0004410	Pentatricopeptide repeat (PPR-like) superfamily protein; (source: Araport11)
ZLC10G0004450	member of SYP12 Gene Family ATSYP124; SYNTAXIN OF PLANTS 124; SYP124
ZLC10G0004460	F-box family protein; (source: Araport11)
ZLC10G0004470	Tetratricopeptide repeat (TPR)-like superfamily protein; (source: Araport11)
ZLC10G0004480	embryo defective 1923; (source: Araport11) EMB1923; EMBRYO DEFECTIVE 1923
ZLC10G0004490	transmembrane protein, putative (DUF707); (source:Araport11)
ZLC10G0004510	NAD(P)H dehydrogenase B1; (source: Araport11) NAD(P)H DEHYDROGENASE B1; NDB1
ZLC10G0004520	diacylglycerol kinase 5; (source: Araport11) ATDGK5; DGK5; DIACYLGLYCEROL KINASE 5
ZLC10G0004550	Encodes a malonyltransferase that may play a role in phenolic xenobiotic detoxification. The mRNA is cell-to-cell mobile. PHENOLIC GLUCOSIDE MALONYLTRANSFERASE 2; PMAT2
ZLC10G0004560	HXXXD-type acyl-transferase family protein; (source: Araport11)
ZLC10G0004580	Encodes a malonyltransferase that may play a role in phenolic xenobiotic detoxification. The mRNA is cell-to-cell mobile. PHENOLIC GLUCOSIDE MALONYLTRANSFERASE 2; PMAT2
ZLC10G0004590	Encodes a MYB transcription factor involved in wounding and osmotic stress response. Member of the R2R3 factor gene family. A.

	THALIANA MYB 4; ATM4; ATMYB102; MYB-LIKE 102; MYB102
ZLC10G0004610	Encodes a nuclear localized aminotransferase-like protein containing a plant mobile domain. The mRNA is cell-to-cell mobile. MAIL2; MAIN-LIKE 2
ZLC10G0004620	Encodes a signalling peptide influencing lateral organ separation. TAX1; TAXIMIN 1
ZLC10G0004640	Member of a large family of putative ligands homologous to the Clavata3 gene. Consists of a single exon. Can replace CLV3 function in vivo. ATCLE6; CLAVATA3/ESR-RELATED 6; CLE6
ZLC10G0004680	Encodes the CHLI subunit of magnesium chelatase which is required for chlorophyll biosynthesis. All four cysteine residues of the protein form two disulfide bonds (Cys102-Cys193 and Cys354-Cys396) under oxidized conditions but are fully reduced by reduction. It was suggested that the redox state of CHLI is regulated in vivo by the change of the redox environment in the chloroplasts probably via the Trx system. CH-42; CH42; CHL11; CHLI-1; CHLI1; CHLORINA 42; LOST1; LOW TEMPERATURE WITH OPEN-STOMATA 1
ZLC10G0004690	Encodes a ppGpp pyrophosphohydrolase. ARABIDOPSIS THALIANA NUDIX HYDROLASE HOMOLOG 15; ATNUDT15; ATNUDX15; NUDIX HYDROLASE HOMOLOG 15; NUDX15
ZLC10G0004700	nudix hydrolase homolog 22; (source: Araport11) ATNUDT22; NUDIX HYDROLASE HOMOLOG 22; NUDT22
ZLC10G0004710	Encodes a ppGpp pyrophosphohydrolase. ARABIDOPSIS THALIANA NUDIX HYDROLASE HOMOLOG 15; ATNUDT15; ATNUDX15; NUDIX HYDROLASE HOMOLOG 15; NUDX15
ZLC10G0004720	Encodes a ppGpp pyrophosphohydrolase. ARABIDOPSIS THALIANA NUDIX HYDROLASE HOMOLOG 15; ATNUDT15; ATNUDX15; NUDIX HYDROLASE HOMOLOG 15; NUDX15
ZLC10G0004760	TCP family protein involved in heterZLConic regulation of leaf differentiation. TCP DOMAIN PROTEIN 10; TCP10
ZLC10G0004770	F-box and associated interaction domains-containing protein; (source: Araport11)
ZLC10G0004780	SGNH hydrolase-type esterase superfamily protein; (source: Araport11)
ZLC10G0004790	Optic atrophy 3 protein (OPA3); (source: Araport11)
ZLC10G0004800	Encodes a homolog of the transcriptional repressor SIN3 (AT1G24190). SIN3-LIKE 2; SNL2
ZLC10G0004810	Enhances AtERF7-mediated transcriptional repression. RNAi lines show ABA hypersensitivity. Interacts with ERF7 and HDA19. SIN3-LIKE 3; SNL3
ZLC10G0004820	Member of IQ67 (CaM binding) domain containing family. IQ-DOMAIN 9; IQD9
ZLC10G0004830	Apoptosis inhibitory protein 5 (API5); (source: Araport11)

ZLC10G0004840	GDSL-motif esterase/acyltransferase/lipase. Enzyme group with broad substrate specificity that may catalyze acyltransfer or hydrolase reactions with lipid and non-lipid substrates.
ZLC10G0004850	GDSL-motif esterase/acyltransferase/lipase. Enzyme group with broad substrate specificity that may catalyze acyltransfer or hydrolase reactions with lipid and non-lipid substrates.
ZLC10G0004860	GDSL-motif esterase/acyltransferase/lipase. Enzyme group with broad substrate specificity that may catalyze acyltransfer or hydrolase reactions with lipid and non-lipid substrates.
ZLC10G0004870	beta glucosidase 17; (source: Araport11) BETA GLUCOSIDASE 17; BGLU17
ZLC10G0004880	GDSL-motif esterase/acyltransferase/lipase. Enzyme group with broad substrate specificity that may catalyze acyltransfer or hydrolase reactions with lipid and non-lipid substrates.
ZLC10G0004890	RAB GTPase homolog A1G; (source: Araport11) ATRABA1G; RAB GTPASE HOMOLOG A1G; RABA1G
ZLC10G0004900	Tail-anchored (TA) OEP membrane protein which possesses a single C-terminal transmembrane domain targeting post-translationally to plastids.
ZLC10G0004910	UDP-Glycosyltransferase superfamily protein; (source: Araport11) UGT79B3
ZLC10G0004920	pentatricopeptide (PPR) repeat-containing protein; (source: Araport11)
ZLC10G0004940	transcription elongation factor-like protein; (source: Araport11)
ZLC10G0004950	The gene encodes a putative nodulin-like21 protein.
ZLC10G0004960	VOZ transcription factor which acts as positive regulator of several salt-responsive genes. Functionally redundant in salt stress with VOZ2. ATVOZ1; VASCULAR PLANT ONE ZINC FINGER PROTEIN; VOZ1
ZLC10G0004970	Optic atrophy 3 protein (OPA3); (source: Araport11)
ZLC10G0004980	Disease resistance-responsive (dirigent-like protein) family protein; (source: Araport11)
ZLC10G0004990	ATP binding microtubule motor family protein; (source: Araport11)
ZLC10G0005000	Member of Kunitz trypsin inhibitor (KTI) family involved in plant defense response against spider mites. ARABIDOPSIS THALIANA KUNITZ TRYPSIN INHIBITOR 5; ATKTI5
ZLC10G0005020	Encodes GRX480, a member of the glutaredoxin family that regulates protein redox state. GRX480 interacts with TGA factors and suppresses JA-responsive PDF1.2 transcription. GRX480 transcription is SA-inducible and requires NPR1. Maybe involved in SA/JA cross-talk. It has also been shown to interact with the transcription factor TGA2 and suppress ORA59 promoter activity. GRX480; GRXC9; ROXY19
ZLC10G0005030	PLAT1 domain stress protein family member. Involved in mediating response to stresses such as pathogen infection. It is found in

	endoplasmic reticulum bodies. PLAT1 is induced by pathogenic fungi and induces the production of scopolin. "ATPLAT1; PLAT DOMAIN PROTEIN 1; PLAT1; POLYCYSTIN, LIPOXYGENASE, ALPHA-TOXIN AND TRIACYLGLYCEROL LIPASE 1"
ZLC10G0005040	Lipase/lipooxygenase, PLAT/LH2 family protein; (source: Araport11) PLAT DOMAIN PROTEIN 2; PLAT2
ZLC10G0005050	Lipase/lipooxygenase, PLAT/LH2 family protein; (source: Araport11) PLAT DOMAIN PROTEIN 2; PLAT2
ZLC10G0005060	Tetratricopeptide repeat (TPR)-like superfamily protein; (source: Araport11)
ZLC10G0005070	Guanylate-binding family protein; (source: Araport11) GBPL3; GUANYLATE-BINDING PROTEIN-LIKE 3
ZLC10G0005080	Protein kinase superfamily protein; (source: Araport11)
ZLC10G0005100	encodes an isoform of 4-coumarate: CoA ligase (4CL), which is involved in the last step of the general phenylpropanoid pathway. In addition to 4-coumarate, it also converts ferulate. The catalytic efficiency was in the following (descending) order: p-coumaric acid, ferulic acid, caffeic acid and 5-OH-ferulic acid. At4CL1 was unable to use sinapic acid as substrate. 4-COUMARATE: COA LIGASE 1; 4CL0; 4CL1; ARABIDOPSIS THALIANA 4-COUMARATE: COA LIGASE 1; AT4CL1
ZLC10G0005110	encodes an isoform of 4-coumarate: CoA ligase (4CL), which is involved in the last step of the general phenylpropanoid pathway. The catalytic efficiency was in the following (descending) order: p-coumaric acid, caffeic acid, ferulic acid, 5-OH-ferulic acid and cinnamic acid. At4CL2 was unable to use sinapic acid as substrate. 4-COUMARATE: COA LIGASE 2; 4CL2; AT4CL2
ZLC10G0005120	Encodes a nuclear cap-binding protein that forms a heterodimeric complex with ABH1 (ATCBP80) and is likely to participate in RNA metabolism. Its mRNA is ubiquitously expressed.Loss of function mutations suggest a role in processing of pri-miRNA and mRNA splicing. ATCBP20; CAP-BINDING PROTEIN 20; CBP20
ZLC10G0005140	Eukaryotic translation initiation factor 3 subunit 7 (eIF-3); (source: Araport11)
ZLC10G0005150	Eukaryotic translation initiation factor 3 subunit 7 (eIF-3); (source: Araport11)
ZLC10G0005160	strawberry notch protein (DUF616); (source: Araport11) MUCI70; MUCILAGE-RELATED 70
ZLC10G0005170	tRNA (met) cytidine acetyltransferase, putative (DUF616); (source: Araport11)
ZLC10G0005180	Tetratricopeptide repeat (TPR)-like superfamily protein; (source: Araport11)
ZLC10G0005190	defensin-like protein; (source: Araport11)

ZLC10G0005200	Concanavalin A-like lectin protein kinase family protein; (source: Araport11) L-TYPE LECTIN RECEPTOR KINASE S.4; LECRK-S.4
ZLC10G0005210	Encodes a sphingolipid delta4-desaturase, involved in sphingolipid biosynthesis. Specifically expressed in floral tissues. Knockout mutants were devoid of sphinga-4,8-dienine in floral tissues. DES-1-LIKE
ZLC10G0005230	RING/U-box superfamily protein; (source: Araport11)
ZLC10G0005240	SGNH hydrolase-type esterase superfamily protein; (source: Araport11)
ZLC10G0005260	Encodes a K (+)/ H (+) antiporter that modulates monovalent cation and pH homeostasis in plant chloroplasts or plastids. ATKEA2; K+ EFFLUX ANTIPORTER 2; KEA2
ZLC10G0005270	Encodes a Golgi apparatus-localized galactosyltransferase involved in galactosyl-substitution of xyloglucan at position 2. ATGT18; GLYCOSYLTRANSFERASE 18; GT18; XLT2; XYLOGLUCAN L-SIDE CHAIN GALACTOSYLTRANSFERASE POSITION 2
ZLC10G0005280	CBS domain-containing protein; (source: Araport11)
ZLC10G0005290	Encodes a protein similar to human SNAP50. Mutants display different temperature sensitivities in the dedifferentiation of cells from different organs. Mutation inhibits the dedifferentiation-associated accumulation of U-snRNAs and some other small RNA species encoded by independent-type genes carrying the USE and TATA box. Required for the elevation of cell proliferation competence in hypocotyl dedifferentiation. SHOOT REDIFFERENTIATION DEFECTIVE 2; SRD2
ZLC10G0005310	Part of multi-protein complex, acting as guanine nucleotide exchange factors (GEFs) and possibly as tethers, regulating intracellular trafficking. TRAPPC13
ZLC10G0005320	H[+]-ATPase 11; (source:Araport11) AHA11; H(+)-ATPASE 11; HA11
ZLC10G0005330	alpha/beta-Hydrolases superfamily protein; (source: Araport11)
ZLC10G0005340	desiccation-like protein; (source: Araport11)
ZLC10G0005350	desiccation-like protein; (source: Araport11)
ZLC10G0005360	desiccation-like protein; (source: Araport11)
ZLC10G0005370	desiccation-like protein; (source: Araport11)
ZLC10G0005380	Encodes a chloroplast triose phosphate / 3-phosphoglycerate translocator that transports triose phosphates derived from the Calvin cycle in the stroma to the cytosol for use in sucrose synthesis and other biosynthetic processes. A tpt mutant has altered acclimation responses. The mRNA is cell-to-cell mobile. ACCLIMATION OF PHOTOSYNTHESIS TO ENVIRONMENT 2; APE2; TPT; TRIOSE-PHOSPHATE ⁄ PHOSPHATE TRANSLOCATOR
ZLC10G0005420	Required for functional maturation of male and female gametophytes. DEAD-BOX RNA HELICASE 29; RH29

Table S6. Mutation of Genes on Chromosome 10

Gene name	Mutation type
ZLC10G0003560	premature termination
ZLC10G0003760	non synonymous mutation
ZLC10G0003800	non synonymous mutation
ZLC10G0003860	premature termination
ZLC10G0003880	non synonymous mutation
ZLC10G0003890	structural variation
ZLC10G0003900	premature termination
ZLC10G0003910	non synonymous mutation
ZLC10G0003920	non synonymous mutation
ZLC10G0003930	frameshift mutation
ZLC10G0003940	non synonymous mutation
ZLC10G0003980	non synonymous mutation
ZLC10G0003990	non synonymous mutation
ZLC10G0004010	premature termination
ZLC10G0004030	non synonymous mutation
ZLC10G0004050	non synonymous mutation
ZLC10G0004060	non synonymous mutation
ZLC10G0004090	non synonymous mutation
ZLC10G0004100	frameshift mutation
ZLC10G0004110	non synonymous mutation
ZLC10G0004130	non synonymous mutation
ZLC10G0004160	non synonymous mutation
ZLC10G0004230	non synonymous mutation
ZLC10G0004250	non synonymous mutation
ZLC10G0004260	non synonymous mutation
ZLC10G0004270	non synonymous mutation
ZLC10G0004300	non synonymous mutation
ZLC10G0004330	frameshift mutation
ZLC10G0004340	non synonymous mutation
ZLC10G0004370	non synonymous mutation

ZLC10G0004390	non synonymous mutation
ZLC10G0004490	non synonymous mutation
ZLC10G0004510	non synonymous mutation
ZLC10G0004520	non synonymous mutation
ZLC10G0004550	non synonymous mutation
ZLC10G0004560	non synonymous mutation
ZLC10G0004580	non synonymous mutation
ZLC10G0004590	non synonymous mutation
ZLC10G0004610	premature termination
ZLC10G0004690	non synonymous mutation
ZLC10G0004710	non synonymous mutation
ZLC10G0004720	non synonymous mutation
ZLC10G0004760	frameshift mutation
ZLC10G0004770	frameshift mutation
ZLC10G0004780	frameshift mutation
ZLC10G0004790	non synonymous mutation
ZLC10G0004800	non synonymous mutation
ZLC10G0004820	non synonymous mutation
ZLC10G0004830	non synonymous mutation
ZLC10G0004850	non synonymous mutation
ZLC10G0004860	non synonymous mutation
ZLC10G0004870	non synonymous mutation
ZLC10G0004880	non synonymous mutation
ZLC10G0004890	non synonymous mutation
ZLC10G0004910	non synonymous mutation
ZLC10G0004920	premature termination
ZLC10G0005020	non synonymous mutation
ZLC10G0005030	non synonymous mutation
ZLC10G0005040	non synonymous mutation
ZLC10G0005060	non synonymous mutation
ZLC10G0005070	non synonymous mutation
ZLC10G0005100	non synonymous mutation
ZLC10G0005120	frameshift mutation

ZLC10G0005140	frameshift mutation
ZLC10G0005150	non synonymous mutation
ZLC10G0005170	premature termination
ZLC10G0005180	non synonymous mutation
ZLC10G0005200	non synonymous mutation
ZLC10G0005240	non synonymous mutation
ZLC10G0005270	non synonymous mutation
ZLC10G0005280	non synonymous mutation

Table S7. DEGs on Chromosome 1

	Gene name
1	*ZLC01G0009030*
2	*ZLC01G0009040*
3	*ZLC01G0009050*
4	*ZLC01G0009060*
5	*ZLC01G0009220*
6	*ZLC01G0009260*
7	*ZLC01G0009380*
8	*ZLC01G0009400*
9	*ZLC01G0009420*
10	*ZLC01G0009530*
11	*ZLC01G0009540*
12	*ZLC01G0009560*
13	*ZLC01G0009600*
14	*ZLC01G0009620*
15	*ZLC01G0009630*
16	*ZLC01G0009640*
17	*ZLC01G0009650*
18	*ZLC01G0009660*
19	*ZLC01G0009680*
20	*ZLC01G0009690*
21	*ZLC01G0009740*
22	*ZLC01G0009760*
23	*ZLC01G0009790*
24	*ZLC01G0009840*

Table S8. DEGs on Chromosome 10

	Gene name
1	*ZLC10G0003560*
2	*ZLC10G0003570*
3	*ZLC10G0003590*

4	*ZLC10G0003620*
5	*ZLC10G0003650*
6	*ZLC10G0003660*
7	*ZLC10G0003680*
8	*ZLC10G0003690*
9	*ZLC10G0003700*
10	*ZLC10G0003720*
11	*ZLC10G0003730*
12	*ZLC10G0003900*
13	*ZLC10G0003980*
14	*ZLC10G0003990*
15	*ZLC10G0004010*
16	*ZLC10G0004060*
17	*ZLC10G0004070*
18	*ZLC10G0004080*
19	*ZLC10G0004090*
20	*ZLC10G0004100*
21	*ZLC10G0004110*
22	*ZLC10G0004160*
23	*ZLC10G0004180*
24	*ZLC10G0004250*
25	*ZLC10G0004260*
26	*ZLC10G0004270*
27	*ZLC10G0004280*
28	*ZLC10G0004310*
29	*ZLC10G0004340*
30	*ZLC10G0004510*
31	*ZLC10G0004580*
32	*ZLC10G0004590*
33	*ZLC10G0004620*
34	*ZLC10G0004690*
35	*ZLC10G0004710*
36	*ZLC10G0004760*
37	*ZLC10G0004770*
38	*ZLC10G0004780*
39	*ZLC10G0004860*
40	*ZLC10G0005000*
41	*ZLC10G0005020*
42	*ZLC10G0005040*
43	*ZLC10G0005050*
44	*ZLC10G0005080*
45	*ZLC10G0005100*
46	*ZLC10G0005110*
47	*ZLC10G0005120*

48	*ZLC10G0005180*
49	*ZLC10G0005210*
50	*ZLC10G0005280*
51	*ZLC10G0005370*

Table S9. Genes related to the color of pepper fruits

Gene name	Function	Relevance to Pepper Color
ZLC01G0009560	Encodes a protein related to nitrate transport (MYB59) [53]	Nitrate metabolism can influence plant metabolic pathways, including pigment biosynthesis, suggesting potential indirect effects on pepper color
ZLC01G0009570	Encodes an R2R3-MYB (MYB111) transcription factor, a family known for its role in regulating anthocyanin and other pigment biosynthesis [54]	The MYB transcription factor family is a well-established regulator of plant pigmentation, and this gene may directly regulate pathways involved in flavonoid and anthocyanin synthesis, potentially influencing pepper fruit color
ZLC01G0009580	Encodes an enzyme involved in carotenoid biosynthesis (ent-kaurenoic acid hydroxylase) [55]	Carotenoids are major pigments responsible for yellow, orange, and red hues in pepper. This gene may contribute to the accumulation of carotenoids in yellow-fruited varieties
ZLC10G0003870	Involved in the metabolism of phenolic compounds, including anthocyanins and flavonoids [56]	This gene could modulate anthocyanin accumulation, influencing red or purple pigmentation in peppers
ZLC10G0003910	Related to oxidative stress response and redox homeostasis [57]	Oxidative stress can influence pigment accumulation, and this gene may have an indirect role in regulating fruit color under stress conditions

ZLC10G0004680	Encodes the CHLI subunit of magnesium chelatase, a key enzyme in chlorophyll biosynthesis	This gene plays a critical role in chlorophyll synthesis, directly influencing the green coloration of pepper fruits
ZLC10G0005100	Encode enzymes involved in the phenylpropanoid pathway, which is critical for flavonoid and anthocyanin biosynthesis [58]	These genes may regulate the synthesis of flavonoid pigments, contributing to variations in fruit color
ZLC10G0005110	Encode enzymes involved in the phenylpropanoid pathway, which is critical for flavonoid and anthocyanin biosynthesis	These genes may regulate the synthesis of flavonoid pigments, contributing to variations in fruit color
ZLC10G0005260	Involved in ion homeostasis within cells [59]	Ion balance can indirectly influence pigment biosynthesis by affecting cellular pH, which is critical for pigment stability
